# Testing pathways to scale: study protocol for a three-arm randomized controlled trial of a centralized and a decentralized (“Train the Trainers”) dissemination of a mental health program for Kenyan adolescents

**DOI:** 10.1186/s13063-023-07539-y

**Published:** 2023-08-13

**Authors:** Brenda Ochuku, Tom L. Osborn, Daisy Nerima, Afra van der Markt, Thomas Rusch, Herman Omune, Solace Akello, David M. Ndetei, Katherine E. Venturo-Conerly

**Affiliations:** 1 Shamiri Institute, Nairobi, Kenya; 2 Shamiri Institute, Allston, MA USA; 3 Amsterdam UMC location VUmc, Psychiatry, Amsterdam, The Netherlands; 4 Competence Center for Empirical Research Methods, WU Vienna University of Economics and Business, Vienna, Austria; 5 African Mental Health Research & Training Foundation, Nairobi, Kenya; 6 Department of Psychiatry, University of Nairobi, Nairobi, Kenya; 7 Department of Psychology, Harvard University, Cambridge, MA USA

**Keywords:** Mental health, Shamiri Intervention, Adolescents, Depression, Anxiety, Character strengths intervention, Psychotherapy, Global Mental Health

## Abstract

**Background:**

Providing care in Kenya to all youth in need is difficult because of a shortage of professional providers and societal stigma. Previous trials of the Anansi model, which involves delivering low-touch mental health interventions through a tiered caregiving model (including lay-providers, supervisors, and clinical experts), have shown its effectiveness for reducing depression and anxiety symptoms in school-going Kenyan adolescents. In this trial, we aim to assess two different scale-up strategies by comparing centralized implementation (i.e., by the organization that designed the Anansi model) against implementation through an implementing partner.

**Methods:**

In this three-arm trial, 1600 adolescents aged 13 to 20 years will be randomized to receive the Shamiri intervention from either the Shamiri Institute or an implementation partner or to be placed in the treatment as usual (TAU) control group. The implementation partner will be trained and supplied with protocols to ensure that the same procedures are followed by both implementors. Implementation activities will run concurrently for both implementors. The Shamiri intervention will be delivered by trained lay providers to groups of 10–15 adolescents over four weekly sessions which will take place in secondary schools in Machakos and Makueni counties in Kenya. The TAU group will receive the usual care offered by their respective schools. Outcomes will be assessed at baseline, midpoint (2 weeks), endpoint (4 weeks), and 1 month follow-up. The analysis will be based on an intent-to-treat approach. Mixed effects models will be used to assess trajectories over time of the primary outcomes (anxiety and depressive symptoms, mental well-being, perceived social support, and academic performance) and secondary outcomes for the intervention groups and the control group. Effect sizes will be computed for the mean differences of the intervention and control arms at midpoint, endpoint, and follow-up.

**Discussion:**

This trial will provide insight into the comparative effectiveness of different strategies for scaling a school-based mental health care model. Findings will also indicate areas for improved efficiency of the model to enhance its replicability by other implementors.

**Trial registration:**

Pan African Clinical Trials Registry (PACTR) (ID: PACTR202305589854478, Approved: 02/05/2023).

**Supplementary Information:**

The online version contains supplementary material available at 10.1186/s13063-023-07539-y.

## Administrative information

Note: The numbers in curly brackets in this protocol refer to SPIRIT checklist item numbers. The order of the items has been modified to group related items (see http://www.equator-network.org/reporting-guidelines/spirit-2013-statement-defining-standard-protocol-itemsfor-clinical-trials/).

**Table:** 

Title {1}	Testing Pathways to Scale: Study Protocol for a Three-arm Randomized Controlled Trial of Centralized and Decentralized Mental Health Program Dissemination for Kenyan Adolescents
Trial registration {2a and 2b}.	PACTR ID: PACTR202305589854478, Approved: 02/05/2023
Protocol version {3}	Protocol version number 1.0 (Date: 15^th^ May 2023).
Funding {4}	Fonds d’Innovation pour le Développement (FID) Grant Number 971_301121
Author details {5a}	1.Shamiri Institute, Nairobi, Kenya & Allston, MA, USA 2. Amsterdam UMC location VUmc, Psychiatry, Amsterdam, The Netherlands 3.Competence Center for Empirical Research Methods, WU Vienna University of Economics and Business, Vienna, Austria 4. African Mental Health Research & Training Foundation, Nairobi, Kenya 5. Department of Psychiatry, University of Nairobi, Nairobi, Kenya 6. Department of Psychology, Harvard University, Cambridge, MA, USA
Name and contact information for the trial sponsor {5b}	Fund for Innovation in Development Email: contact@fundinnovation.dev
Role of sponsor {5c}	This study was funded by the Fund for Innovation in Development, located at 5, rue Roland Barthes 75 598 PARIS CEDEX 12 FRANCE. The study design; data collection, management, analysis, and interpretation; writing the report; and the decision to submit the report for publication are the authors’ sole responsibilities.

## Introduction

### Background and rationale {6a}

Mental disorders remain severely undertreated around the world. The World Health Organization (WHO) highlights that mental disorders are widely misunderstood, underfunded, and under-resourced in all countries and more severely so in low- and middle-income countries (LMICs) [1, 2]. Due to inadequate treatment, mental disorders are the second leading global cause of disability [3]. From 1990 to 2019, the global disability-adjusted life years (DALYs) burden attributed to mental disorders increased by 58.1% to 125.3 million [4].

Increased symptoms of anxiety and depression are highly prevalent among adolescents in Sub-Saharan Africa (SSA) [2, 5]. Early identification and treatment of mental disorders are critical to prevent risky behaviors, social and health risks, and over-institutionalization [2, 6]. However, limited access to mental health care in these regions hinders young people’s ability to lead healthy and fulfilling lives [5]. The scarcity of trained mental healthcare providers, lack of mental health resources, societal stigma, and lack of culturally appropriate interventions are barriers young people in SSA face to accessing mental health services [7–10]. Therefore, there is a need to develop and evaluate scalable and low-cost mental health interventions.

To address this gap, researchers have tested several mental health interventions for youth living in low- and middle-income countries [11], including the Shamiri Intervention, a brief character strengths intervention that has been tested in several trials [10]. The Shamiri (Swahili for “thrive”) intervention [8, 12, 13] was designed to improve mental health and wellbeing by shifting young people’s perceptions of themselves and their world. It is administered by lay providers in four hourly sessions across four weeks and consists of three modules: growth mindset, value affirmations, and gratitude [6, 14, 15]. The intervention has demonstrated promising outcomes in enhancing academic performance, psychosocial well-being, and mental health among adolescents. These results add to the growing body of literature on brief low-touch interventions for youth mental health outcomes [15, 16].

The initial randomized controlled trial of the Shamiri intervention was implemented among school-going Kenyan youths with elevated depression and anxiety symptoms (*n* = 51). Compared to an active study skills control group, the intervention significantly improved participants’ depressive and anxiety symptoms, perceived social support, and academic performance [12]. These findings were replicated in a pre-registered study with a larger sample of participants (*n* = 413) with the intervention’s effects persisting at the 7-month follow-up [8]. The development and evaluation of the Shamiri intervention demonstrate the potential for character strength interventions to improve the mental health, academic, and social outcomes of Kenyan youth.

A question that emerges is how do we scale up these types of interventions to address the youth mental health treatment gap? One way of doing this is developing strategies that can allow for these light-touch, school-based interventions, like the Shamiri intervention, to be delivered through possibly scalable models. Our team sought to develop such a model for the delivery and scaling of the Shamiri intervention. The model that we developed was a three-tier caregiving model consisting of mental health workers with varying levels of training [17]. At the first tier of the model are lay providers; recent high school graduates who receive training to lead group sessions. The second tier consists of clinical supervisors who have some mental health experience (e.g., a bachelor’s degree in psychology) and are further trained through a 7-week program that covers topics including supervision and support for lay providers, handling clinical emergencies, providing one-on-one support to students, and referring participants to the third (expert) tier of the model when necessary. The third tier is composed of a clinical network of Kenyan mental health experts, most with doctoral degrees in psychology or psychiatry [12, 17]. This model, which is sometimes referred to as the “Anansi” model, allows for the expansion of the caregiving supply by mobilizing an expanded array of caregivers and offers opportunities for clinical escalation and triaging [18]. Over the past 3 years, more than 9500 youths have received the Shamiri intervention through the Anansi model, demonstrating the model’s scalability [19, 20].

We use two ways of scaling up mental healthcare models. The first, which we call a “centralized approach,” involves an organization ramping up its internal capabilities to increase the reach of its model. In this approach, scale-up is done entirely by the organization through which an intervention is developed. The second approach, which we call a “decentralized” approach (and is sometimes also called a “train-the-trainers” approach), involves an organization training other third-party organizations to replicate its own model.

This study builds on the promising findings on the Shamiri intervention and tests the two possible pathways of scaling up the Shamiri intervention. Particularly, we aim to evaluate whether implementation of the Shamiri intervention through the Anansi model retains its effectiveness in improving youth psychological, social, and academic outcomes when implemented at scale by an external partner through the decentralized scale-up model. Additionally, this trial will shed light on the strengths and limitations of the Anansi model for scaling up.

### Objectives {7}

The primary objective of this study is to explore ways of scaling the Shamiri intervention through the Anansi model in Kenyan secondary schools. We will realize this aim through two specific objectives. First, this study will test the effectiveness of the Anansi model in school settings through a large-scale dissemination study with 1600 adolescents. We hypothesize that the Shamiri intervention will positively influence adolescent’s psychological, social, and academic outcomes when delivered through the Anansi model. The second objective is to compare two strategies of scaling the Shamiri intervention through Anansi in Kenya, either implemented by the Shamiri Institute or by a community-based partner. We hypothesize that the intervention run by the community-based partner will be equally effective as the one carried out by the Shamiri Institute.

### Trial design {8}

To assess the effectiveness of two different approaches for scaling the Anansi model of tiered lay psychotherapy, we will conduct a three-arm randomized controlled trial in seven Kenyan secondary schools. The Shamiri Institute team will partner with a community-based implementing partner, Africa Mental Health Research and Training Foundation (AMHRTF), to trial the Anansi care-giving model as an after-school program. We will train the implementation partner and provide them with a manual and protocol to ensure that both parties follow the same implementation procedures. The partner will implement the intervention in parallel with the Shamiri Institute.

Throughout this protocol, the delivery of the Shamiri intervention by the Shamiri Institute is referred to as the centralized approach whereas delivery of Shamiri intervention through the implementing partner is referred to as the decentralized approach. To compare the effectiveness of the centralized and decentralized approaches, participants will be randomized to receive the Shamiri intervention either through the Shamiri Institute or the community-based implementing partner or be randomized to a treatment as usual (TAU) control group.

## Methods: participants, interventions, and outcomes

### Study setting {9}

This study will be conducted in seven high schools across two out of the forty-seven Kenyan counties, namely Makueni and Machakos. Makueni County has a population of around 990 thousand, while Machakos County has about 1.4 million [18]. The study team has extensive experience working with schools in the targeted counties [8, 21]. In Kenya, the Ministry of Education categorizes high schools in three broad categories: private, Harambee, and state-owned schools. Private schools are funded by individuals or private organizations, Harambee schools are built and funded by the community, and state-owned schools are funded by the government. The schools are further classified as either national, extra-county, county, or sub-county schools—and as day, boarding, girls only, boys only, or mixed-gender [21].

National schools are for students with particularly high-test scores, while extra-county schools are ranked after national schools. Students who do not qualify for these schools may be placed in county schools, which are ranked higher than sub-county schools. We aim to include a diverse set of schools that represent the nature of schools in Kenya while prioritizing adolescents in lower-resource settings (e.g., prioritizing public over private schools) since the Shamiri intervention is primarily aimed to be used in a lower-resource setting.

## Eligibility criteria {10}

### Participant inclusion and exclusion criteria

To enroll in the study, school administrators will be invited to sign a memorandum of understanding (MoU) indicating their willingness to participate. The students from the participating schools will be informed about the study by a contact person from the school. Then, the project team will organize a session with students one week before implementation starts to describe the program in detail. All students aged 13 to 20 who express interest in joining the program will be eligible for the study, with no additional exclusion criteria applied.

The Shamiri intervention will be delivered by trained group facilitators, all lay providers with at least a high school degree, who will each lead the intervention for a group of 10 to 15 adolescents. Lay providers will be included if they live near Makueni or Machakos counties, are between the ages of 18 and 24, and graduated from a Kenyan secondary school. Applicants who meet these criteria will participate in 30-min interviews and will be selected based on their counseling or group facilitation skills and knowledge of common issues faced by Kenyan secondary school students. The recruitment and training of lay providers will closely mirror the procedures employed in previous trials of the Shamiri Intervention [17].

Recruiting local peers as lay providers has several advantages; the local lay providers are connected and well-versed in the culture of our target population and will thus be able to relate well to the target group. Furthermore, if the intervention again proves to be successful, one of the most efficient and cost-effective ways of scaling up will be through local lay providers.

### Who will take informed consent? {26a}

Local customs and policies authorize school administrations to decide on any activity (including research) involving students. Therefore, the school heads will represent guardians and parents in receiving information about the study and may contact parents and guardians according to local customs [22]. The school heads will be given the opportunity to ask questions concerning the study and provide consent for all students by enrolling their school into the program by signing an MoU. The school administrators will be given an option to contact the students’ parents and guardians at home if they deem it appropriate.

Also, before sessions begin, interested students will be provided with more information about the study and an opportunity to ask questions. Students will be informed that participating in the study is voluntary and that they can opt out of the study at any time. Interested students will then be asked to provide consent, or assent for minors, prior to enrollment in the study.

### Additional consent provisions for collection and use of participant data and biological specimens {26b}

N/A. We will only collect the data mentioned in the “Outcomes {12}” section, and no biological specimens will be collected in this study.

## Interventions

### Explanation for the choice of comparators {6b}

The primary objective of this trial is to compare a centralized vs decentralized approach to scaling, assessing the comparative effectiveness of each approach. To achieve this, the Shamiri-Institute-delivered intervention (centralized) will be compared against the Shamiri Intervention delivered by the implementing partner (decentralized). A treatment as usual control group is included to provide a practical reference point from which the intervention’s efficacy for both implementors will be estimated relative to what school-going adolescents would typically receive.

## Intervention description {11a}

### Shamiri intervention

The Shamiri intervention entails four weekly one-hour group sessions. The first two sessions are centered around growth mindset. The third and fourth sessions focus on gratitude and value affirmation, respectively. Sample intervention protocols can be found in supplementary materials.

#### Session 1: Growth mindset

This first session will introduce adolescents to the concept of having a growth mindset [23, 24] which is defined as the belief that intelligence, abilities, and talents can be developed through hard work, persistence, and dedication. Adolescents will discuss the human capacity for personal improvement and growth in various areas of their lives including emotional, social, and academic.

Additionally, students will be introduced to the concept of neuroplasticity, the idea that the human brain grows and makes new connections by learning and practicing new skills, through a short script. As part of the session, adolescents will read growth testimonials from local Kenyan peers that cover personal growth in various areas including social life, personality, academic performance, happiness, and views. At the end of the first session, students will receive an assignment that asks them to reflect on a challenge they have faced, strategies they used to address it, and reflect on how the challenge influenced their personal growth. This take-home assignment will be discussed during the following week.

#### Session 2: Growth mindset

The second session will entail discussing the take-home assignment, a letter-writing exercise, and completing a problem-solving practice task. The session will begin with the participants sharing feedback on the assignments they completed during the previous week. The students will then engage in a letter-writing exercise, where they will write a letter to a friend explaining what they have learned so far, including problem-solving, effective strategies, growth mindset, and neuroplasticity.

At the end of session two, the study participants will be given an assignment focused on problem-solving. They will be asked to identify one problem they are facing at that time and apply the problem-solving skills they learned to come up with a solution. They will be encouraged to implement one or two solutions during that week.

#### Session 3: Gratitude

During the third session, participants will be introduced to gratitude and its benefits [20–22]. They will have the opportunity to discuss this concept and mention the people and things in their lives for which they are grateful. The participants will then write a “gratitude letter” to a person who has positively impacted their life.

At the conclusion of this session, participants will be given an assignment to write down three positive things from their lives. They will be instructed to carry out this exercise daily and write a brief reflection for each item listed in the daily exercise.

#### Session 4: Value affirmation

The fourth session will be focused on value affirmation [25, 26]. In the Kenyan context, the term “virtue” is often used interchangeably with “value” [13, 20]. The lay provider will begin the session by leading a discussion on the concept of values/virtues and why they are important in shaping purpose and behavior.

From a long list of values/virtues, the group members will be asked to pick the ones that are most meaningful to them. They will then be asked to write on the importance of the selected value/virtue, how they have demonstrated it in the past, and how they will incorporate it in the future and connect it to their personal goals. At the end of this session, no additional assignments will be given to the participants.

### Treatment as usual control

Participants who are assigned to receive treatment as usual will receive the standard care that is offered at their respective schools [27]. Schools in Kenya offer different forms of care to students in need ranging from guidance and counseling from teacher to community activities and support. Besides this, students in the TAU control group will be allowed to approach the study team members at any time with questions and concerns. Students who need urgent support will be attended to according to our emergency protocol and will not be excluded from participating unless it is deemed necessary for their safety and wellbeing.

### Criteria for discontinuing or modifying allocated interventions {11b}

A participant may discontinue their assigned intervention if they decide to withdraw from the intervention, exhibit behavior so disruptive that it harms the other participant’s ability to participate even after requests to change their behavior or if the study team determines that it is necessary for the participant’s safety and well-being for them to cease participating. We expect some students to voluntarily choose to leave the study, but we expect there will be minimal or no need to forcibly remove any participants. If a participant misses any session, they will still be allowed to participate in the study. Participants will be encouraged to maintain the allocated groups throughout the study with no modifications or discontinuations.

Once the trial commences, we do not intend to modify the assigned interventions. However, prior to the start of implementation, minor adjustments may be made to the intervention protocols (e.g., phrasing edits) based on feedback from trainees or other stakeholders. The final intervention protocols will be made available in published manuscripts.

### Strategies to improve adherence to interventions {11c}

We will employ several strategies to encourage and evaluate compliance with intervention procedures. The first involves comprehensive training for all lay providers. They will undergo didactic training and engage in roleplays to effectively administer the intervention. They will also be instructed on the use of the emergency protocols when necessary and will be instructed in and expected to follow general trial guidelines, such as maintaining participant confidentiality. This training will be supplemented by tailored assessments such as a post-training readiness survey and real-time role play evaluation and feedback.

Second, we will ensure adherence through supervision. This will include both weekly in-person meetings and daily supervision by trained supervisors to verify compliance with the intervention protocols. All supervisors will have at least a bachelor’s degree in psychology and prior counseling experience. They will undertake a seven-week training on ethical considerations, the Shamiri Intervention, and trial protocols. Furthermore, a clinical network consisting of PhD-level mental health experts will oversee these supervisors.

The final approach is the measurement of adherence to the protocols during intervention implementation. This will be accomplished by enlisting independent raters to assess a randomly selected portion (10%) of session recordings on fidelity and performance, using a method similar to previous trials [8, 13, 20]. Each recording will be evaluated by two independent raters, after training on the use of the fidelity rubric, and their level of agreement will be assessed using Gwet’s AC2 [28, 29] for ordinal rating scales.

### Relevant concomitant care permitted or prohibited during the trial {11d}

During the trial, participants will have the freedom to seek alternative forms of care or use medication. Those in the control condition will be encouraged and referred to utilize the standard mental health services provided by their respective schools. However, as mentioned in the “Adverse event reporting and harms {22}” section, participants identified by the study team as presenting significant risk of self-harm or harm to others will be strongly encouraged to seek professional mental health assistance from a provider from the network of mental health experts affiliated with the study team. If a student requires ongoing post-trial care, arrangements will be made in coordination with the school administrators. Unless the principal investigator (PI) deems it necessary for the participants’ well-being, students referred to external care will not be excluded from the sample or removed from the groups.

### Provisions for post-trial care {30}

As explained in the “Adverse event reporting and harms {22}” section, the study team will periodically evaluate the level of risk of participants who exhibit potential risk of self-harm or harm to others using the emergency protocol (refer to the supplementary materials for the complete emergency protocol). If deemed necessary by the study team, these students will be referred to the clinical network, the third tier of the Anansi caregiving model, for individual support both during or after the trial. We do not anticipate any harm resulting from participants’ involvement in the trial, and as a result, we have no provisions for compensation in case of harm.

## Outcomes {12}

### Primary outcomes

#### Patient Health Questionnaire-8 (PHQ_8)

We will use the PHQ-8 to assess adolescents depression symptoms. PHQ-8 is a shortened version of PHQ-9 that excludes the ninth suicidal ideation item at the advice of Kenyan stakeholders [30, 31]. PHQ-9 and PH-8 measures are highly correlated, and the same cutoffs can be used to assess depression severity. We have previously validated PHQ_8 among Kenyan adolescents and it showed good internal consistency (Cronbach’s alpha = 0.78) [8, 31–33].

#### Generalized Anxiety Disorder-7 (GAD_7)

The GAD-7 is a globally used measure for screening generalized anxiety disorder in both adolescents and adults. GAD-7 has been validated for use in Kenyan adolescents (Cronbach’s alpha = 0.82) [8, 32, 33].

#### Multidimensional Scale of Perceived Social Support (MSPSS)

The MSPSS is designed to assess satisfaction with social support. It is composed of three subscales: the “friends” subscale, the “family” subscale, and the “significant others” subscale. The MSPSS has demonstrated adequate internal consistency in Kenyan adolescents (Cronbach’s alpha = 0.86) [12, 32, 33]. In this study, we will use the friends and family subscales to evaluate adolescents perceived social support.

#### Academic performance

Data on academic performance will be collected for the school term preceding, during, and after study implementation. We will measure students’ academic performance by calculating their average grade across all subjects each semester. Grades will be standardized using a method utilized in previous trials [12] to allow comparison between schools and across different grade levels.

#### Short Warwick-Edinburgh Measure of Mental Well-Being Scale (SWEMWBS)

The SWEMWBS is a shortened version of Warwick-Edinburgh Measure of Mental Well-Being Scale (WEMBS). Unlike the WEMBS, which has 14 items, SWEMBS has 7 items, and it is used to assess an individual’s general mental well-being. This measure has been used in past studies [8, 13, 32] in Kenya and has demonstrated good psychometric properties (Cronbach’s alpha = 0.70) [34].

### Secondary outcomes

#### Perceived Control Scale (PCS) Academics

The PCS Academics consists of six items related to beliefs about personal control, specifically the belief that one can obtain desired outcomes and avoid undesired outcomes in school through effort. PCS has demonstrated adequate internal consistency among Kenyan adolescents (Cronbach’s alpha = 0.78) [32, 33].

#### The Gratitude Questionnaire-6 (GQ_6)

The GQ_6 is a six-item questionnaire that assesses individual experiences of appreciation and gratitude in daily life. The GQ has been validated in Chinese and Taiwanese populations with Cronbach’s alpha ranging from 0.76 to 0.84 [35, 36] for the six items. We have used GQ_6 in our past studies with Kenyan adolescents, and it showed good internal consistency (Cronbach’s alpha = 0.79) [32]. We will use it to assess participant’s gratitude and appreciation experiences in this trial.

#### School Engagement Scale (SES)

We will use SES to assess cognitive, emotional, and behavioral characteristics of students’ school engagement. This scale has been validated in Chicago, India, Turkey, and Russia, showing acceptable internal consistency with a Cronbach’s alpha of 0.72–0.86 [37–40]. Additionally, the SES scale has also been used in South Africa to predict school engagement and it showed excellent internal consistency (Cronbach’s alpha = 0.92) [41]. Additionally, the SES scale has also been used in South Africa to predict school engagement and it showed excellent internal consistency (Cronbach’s alpha = 0.92) [41].

#### Demographics

We will collect socio-demographic information from the participants, including age, form, gender, county, socio-economic status, family, home, and religion/spirituality using a self-report questionnaire (see supplementary materials).

### Participant timeline {13}

The study will be implemented between May and August 2023. During this period, data will be collected within specified periods: at baseline (0 weeks into the study), midpoint (2 weeks into the study), endpoint (4 weeks into the study), and 1-month follow-up (8 weeks into the study) as shown in Table 1.

**Table 1:**
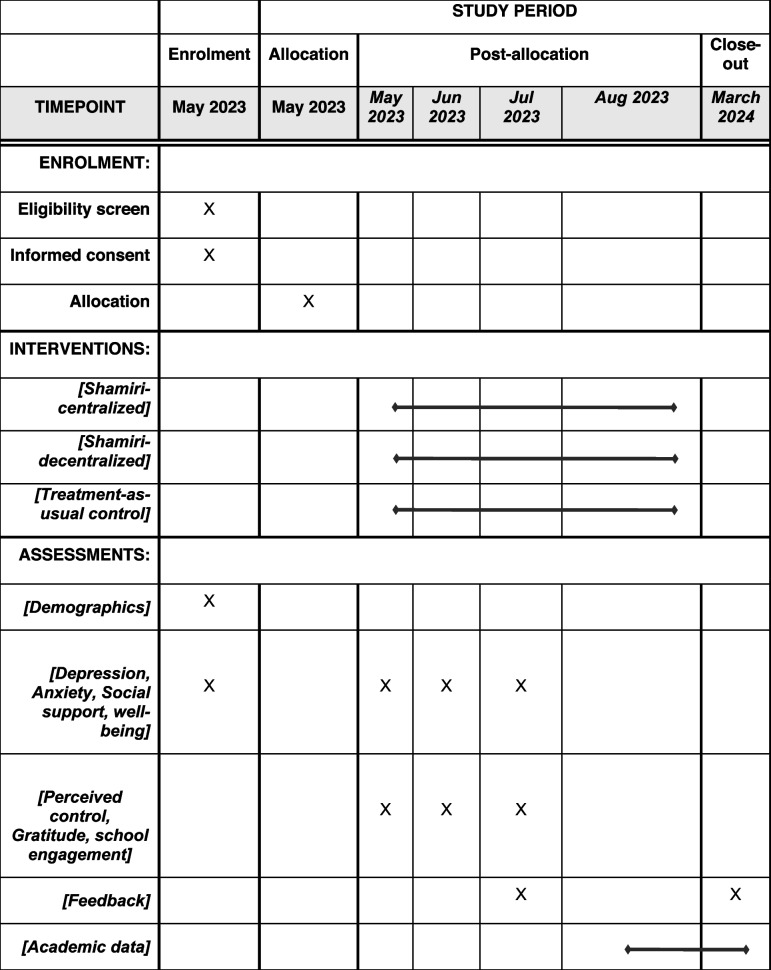
Schedule for enrollment, interventions, and outcomes for the three-arm trial for scaling Anansi model

### Sample size {14}

We used the *simr* package in R to estimate the sample size needed for this trial. We used the lowest standardized effect size between the intervention and control we observed in a prior study [8] as our target effects, which was 0.22. Power simulations revealed that to detect an effect size of at least 0.22 at any time point with a significance level of *α* = 0.05 and a power of at least 80%, an effective sample size (i.e., including attrition of 40%) of 1554 participants is required. We expect up to 30% attrition at the midpoint and endpoint and up to 40% by follow-up based on our past trial [8], which is high. We rounded this up to 1600 participants required at baseline. For more details on our sample size determination and power analysis procedure, please see the supplementary materials.

### Recruitment {15}

Our target population is Kenyan adolescents aged 13 to 20 years who are currently in high school. We aim to recruit 25 schools with a mix of boarding, day, girls-only, boys-only, and mixed-gender schools. The schools we target will be either county, extra-county, or sub-county schools. Heads of the targeted schools will be contacted and informed about the study, after which they will be asked to sign a Memorandum of Understanding (MoU) and provide a contact person from the school to work with us. From the list of schools that sign up for the program, we will select seven schools to create a sub-sample of 1600 students. This will take into consideration the classification of the school (i.e., county, extra county, boy-only etc.), the school population, the school location, and other logistical considerations (e.g., ensuring there are enough lay providers to facilitate sessions on the days proposed by schools). We will include a certain number of students from each school in the sample to ensure that we have 1600 students in total, with an equal gender split. Those in the selected sample schools who are not included in the sample will receive intervention, however will not complete any data collection measures.

## Assignment of interventions: allocation

### Sequence generation {16a}

Consenting students will be randomized into one of the three intervention arms using “ballot boxes.” Each supervisor will prepare a “ballot box” containing small pieces of paper which will indicate the group numbers and the intervention arm assigned. Participants will be invited to line up and pick a folded piece of paper from inside the ballot box, which will indicate their group number and allocated intervention. We aim to have equal numbers of students in each randomized group.

### Concealment mechanism {16b}

The numbers inside the ballot boxes will be folded and thus concealed when the participant picks their number, preventing them from influencing their group assignment. This approach guarantees an equal chance for each participant to be placed in any of the groups.

### Implementation {16c}

Once students have been given their group numbers, they will go to their assigned groups to meet their group leaders, who will then confirm that they are in the correct group. Once the participant’s settle in their groups, the group leader will direct the filling of an attendance sheet containing participant information—name, admission number, and form—then randomization will be complete. The specific groups and arms assigned will be maintained throughout the study.

## Assignment of interventions: blinding

### Who will be blinded {17a}

Study participants, lay providers, and supervisors cannot be blinded to treatment allocation; however, they will not be informed about the study objectives. They will only be informed that the study is out to test different programs to better understand their impact on academic scores and wellbeing.

Additionally, the school administrators will not know the participants’ response to any of the questions and the participants will be assured at the beginning of the sessions that their responses will be kept confidential.

### Procedure for unblinding if needed {17b}

Unblinding will only be needed in the event of a safety concern. In such situations, the team responsible for direct implementation (that is, the lay provider, supervisor, and clinical expert) will need to know the participant’s identity and assigned group. Unblinding procedures will only be required for the people interacting directly with the participants in case an emergency case comes up.

## Data collection and management

### Plans for assessment and collection of outcomes {18a}

One member of the study team will lead the production, distribution, collection, and entry of study questionnaires. Centralizing this process will allow quick identification and resolution of issues. The questionnaire will contain the tools mentioned in the “Outcomes {12}” section.

To ensure data quality, we will thoroughly train relevant actors on data handling procedures—we expect the lay providers to guide students as needed while filling the questionnaires. Members of the study team will be present and accessible virtually during the entire process of data collection, and supervisors onsite will assess lay-providers adherence to protocol. We will provide our implementation partner with a detailed manual on all procedures involved— they will be responsible for their entire process of data collection at their assigned sites to provide an accurate picture of the dynamics involved in decentralization.

### Plans to promote participant retention and complete follow-up {18b}

In the participating schools, the study team will meet all the students and hold a presentation about the program during a pre-session 1 week before implementation begins. One teacher will work closely with the study team as the contact person. We will collaborate to create schedules consistent with the schools’ calendars and we will continuously communicate with teachers during the implementation and at follow-up.

Also, we will consistently monitor attendance rates and audio recordings from the weekly sessions. The supervisors will receive attendance data from the lay providers after every session and feed the data into our real time dashboard. The study team will continuously monitor the session attendance and strategize better to promote participant retention throughout the study.

### Data management {19}

We will collect the questionnaires, attendance sheets, and recorders from each lay provider and ensure that they are stored in a safe and enclosed location. The physical questionnaires will be stored in a container and locked. This container will be transported to the coordination site headquarters, for data processing.

We will extract data from the paper questionnaires using *PaperSurvey* and store it in a password-protected database on *Airtable.PaperSurvey* is an online service that transforms handwritten surveys into digital data with AI recognition technologies while *Airtable* is a cloud-based spreadsheet-database hybrid.

### Confidentiality {27}

We will assign random ID numbers to protect the students’ identities. The questionnaires will be entered in a private setting, kept in a locked cabinet, and destroyed after we have extracted all the data. Only the study team will have access to these materials. No information that may reveal the participants’ identity will be released or published without their consent.

### Plans for collection, laboratory evaluation, and storage of biological specimens for genetic or molecular analysis in this trial/future use {33}

N/A. We will not collect any biological specimens.

## Statistical methods

### Statistical methods for primary and secondary outcomes {20a}

Our data analysis plan will involve descriptive statistics, visualizations, and models of change over time. We will follow an intent-to-treat approach [41], analyzing all the study participants assigned to their intervention arm. Our findings will be summarized using tables and graphs.

For descriptive statistics, we will compute the frequencies and percentages of the demographic and clinical characteristics for the study participants at baseline by the intervention arm. To test for significant group differences in the sample characteristic variables, we will use *F*, *t*, and *χ*^2^ statistics [42].

Primary outcome variations across all the time points will be visualized using multiple plots. We will include line plots for depressive symptoms, anxiety symptoms, academic grades, well-being, and perceived social support for the Shamiri intervention per implementer and TAU control arm, visualizing scores before, during, and after the intervention and control condition.

We will build linear mixed effects models [43, 44] as part of the analysis to compare the outcomes of the two Shamiri intervention and the TAU control groups. These models will consider the hierarchical nature of the data, where participants are nested within organizational structures (participant in lay provider/group in school in county). The standard models will be random-intercept models; we will also test the inclusion of random slopes for individual differences. Some of the model elements (e.g., multiple levels of nesting and the random slope) may result in models that are too complex, unidentifiable, or overfit; in such a case, more parsimonious models will be selected.

The arm, time, and interaction between these two will be included in all the models as fixed effects. Other variables that will be included either in a fixed effect or in a random effect specification in the models are age, gender, lay provider, lay provider gender, school, and county. Baseline scores may also be included. Age will be included as a covariate as older adolescents in Kenya have been found to on average have increased psychosocial stress compared to younger adolescents which may heighten depressive and anxiety symptoms [12].

Gender will be included because Kenyan adolescents have shown gender differences in internalizing problems [8, 12]. Female adolescents have previously reported higher anxiety and depression symptoms as compared to males. Past studies [8, 12] have shown that adolescents in lower-resource schools report higher depression and anxiety symptoms. Lastly, we will include details of lay providers like gender because we would like to explore if the gender of a lay provider affects the study participants.

A *p*-value less than 0.05 in the interaction between the time variable and the arm variable will be considered statistically significant. If the interaction is significant, we will look at the conditional mean difference between the arms at each time point from end point to follow-up. We will assess this and report marginal (predictive) means. If these differences are significant (*p* < 0.05) and the control has scores associated with less favorable outcomes, we will conclude there was improvement due to centralized or decentralized arm over time. If the interaction is not significant but the main effect of centralized or decentralized arm is, and the effect points to lower symptoms in the centralized or decentralized arms, we conclude there was improvement due to centralized or decentralized delivery.

Effect sizes (ESs) will be computed from the mean differences across the different time points for the arms. We will compare Cohen’s D for each outcome at baseline, midpoint, endpoint, and 1-month follow-up for the study participants in the Shamiri intervention arms and TAU control arm. Statistically significant Cohen’s *D* in the direction of less symptoms on average in the arms will reflect greater improvements for intervention compared to control.

### Interim analyses {21b}

N/A. We have no plans to conduct any analyses during the intervention sessions, as the sessions span only a 4-week period. Furthermore, we have not established any stopping guidelines as we expect no harm to the participants because of our study. However, if our tiered-model team comes across a participant who faces an elevated risk, the clinical network may choose to withdraw that participant from the study and offer more intensive clinical support instead. Refer to the supplementary emergency protocol for more details about the emergency protocol and risk assessment.

### Methods for additional analyses (e.g., subgroup analyses) {20b}

N/A. We do not have specific plans for any additional analyses based on subgroup or adjusted analyses besides the main analyses, though some may be conducted in an exploratory manner and reported as such. We do, however, plan to carry out moderator and mediator analyses which will not be part of the primary and secondary outcomes paper.

### Methods in analysis to handle protocol non-adherence and any statistical methods to handle missing data {20c}

We strive to reduce the percentage of missing data and limit attrition as much as possible. Should missing data, we will handle them statistically in the following way: missing data will be imputed five times using the multivariate imputation by chained equations (MICE) [44] algorithm with predictive mean matching in R; predictive mean matching appears to, at least in some cases, produce the least biased estimates and better model performance measures than other imputation methods [45].

As mentioned in the “Strategies to improve adherence to interventions {11c}” section, group sessions will be recorded and rated by trained independent evaluators. We plan to monitor these ratings in real time. In case systemic deviations from the protocol are identified in the sessions, the lay provider will be notified by the supervisor and asked to refer to the manual before the next session. Lay providers who deviate from the protocol will be continuously monitored in the next sessions. If any lay provider exhibits notable non-adherence to the protocol in more than three sessions, we may have to exclude the data associated with the students who would have been trained by these lay providers for sensitivity analysis.

### Plans to give access to the full protocol, participant-level data, and statistical code {31c}

The full protocol and data and statistical code used for analysis will be available online via Open Science Foundation. The data will be cleaned and de-identified before it is shared with the public.

## Oversight and monitoring

### Composition of the coordinating center and trial steering committee {5d}

The trial steering committee is comprised of the PI (principal investigator) and a research management team. The research management team is responsible for overseeing the trial—including recruiting participating schools and students, obtaining consent, and providing support for lay providers and research assistants among other day-to-day study activities. The PI will supervise the research management processes of the research management team. Our implementing partner will have a similar structure.

### Composition of the data monitoring committee, its role and reporting structure {21a}

N/A. We shall not have a data monitoring committee.

### Adverse event reporting and harms {22}

Our network of expert clinicians will lead processes involving adverse events and harm. To assist them, the study staff will complete emergency protocol training before we start implementation. This protocol focuses on Substance Abuse Risk, Violence/Abuse Risk and Suicide/Self-harm Risk events, offering guidance on how to rank risk levels in each, i.e., no risk, low risk, medium risk, high risk. This training will guide preliminary assessment of potentially harmful or adverse events. In cases where harm may be imminent or risk of harm is high, we will always inform the guardian of youth risk; however, in cases in which the harm is not intended or imminent, steps are less clear-cut and must involve balancing participant comfort and confidentiality with safety—safety will take precedence. We will require study staff to notify our network of expert clinicians of all cases being monitored for potential risk.

### Frequency and plans for auditing trial conduct {23}

N/A. We do not have any plans for auditing trial conduct because our study procedures and methods are less likely to affect the participants. If the sponsors, Institutional Review Board (IRB), or investigators raise concerns regarding any aspect of the study, we will invite an independent auditor who will aid in diagnosing and resolving the issues raised.

### Plans for communicating important protocol amendments to relevant parties (e.g., trial participants, ethical committees) {25}

First, any protocol amendments will be communicated to the investigators through conference meetings, emails, or phone. The investigators will be required to judge the proposed amendments. Second, the Kenyatta University IRB will also be notified of important changes via email and through yearly study updates, and we will communicate publicly study updates through the online PACTR. Once verified by the IRB and investigators, we will notify our trial participants as necessary about the changes through meetings in their respective schools.

### Dissemination plans {31a}

We will write a school report highlighting the baseline characteristics of the students in aggregate at each of the schools we visit. The study team will present this report to the participating schools at the end of study. Moreover, we plan to publicly share publications on the baseline characteristics, implementation strategies, cost-effectiveness, mental health, and well-being outcomes of Kenyan adolescents. We will strive to make all published articles open access.

## Discussion

In LMICs such as Kenya, the mental health treatment gap is estimated to be as high as 85%. Addressing this treatment gap has become an urgent global health priority, for which interventions need to be developed and implemented. Recent evidence highlights the potential of brief, low-touch interventions that focus on overall human functioning rather than solely targeting psychopathology [11, 46]. Within this context, the Shamiri intervention has been developed as a school-based and a lay-provider delivered intervention targeting anxiety and depression symptoms in adolescents. Previous RCTs of the Shamiri intervention have demonstrated its effectiveness in reducing depression and anxiety symptoms, with effects comparable to those of traditional psychotherapy [8].

In addition to reaching adolescents that otherwise would not have access to mental healthcare, this type of intervention also has the potential to reduce stigma as students are treated equally in a school-based group setting regardless of their symptom levels and the intervention may eliminate the need to travel to a clinical setting for treatment. However, to reach as many youths as possible, the Shamiri intervention needs scaling. It is still not known whether the effectiveness of the intervention is maintained when scaling up the intervention. To address this knowledge gap, we have here detailed a study protocol that outlines a trial of two possible methods of scaling up this intervention: through centralized scaling and through decentralized (“train the trainers”) scaling where an external partner delivers the Shamiri intervention. This is one of the few ways to further increase the reach of the Shamiri intervention.

If this trial successfully demonstrates that the intervention maintains its utility irrespective of the implementation partner, it would provide valuable support for scaling up the intervention through an implementation partner, which would expand the reach of this low-cost meaningful and impactful intervention which can play a role in addressing the mental health treatment gap. This could mean an improvement of mental health for the adolescents themselves, but also an improvement for the school as a whole, by creating a positive learning environment and improving academic performance.

Besides the many strengths of the study, there may also be several limitations. Primarily, all students are eligible to participate regardless of their symptoms level. While this approach has advantages such as not requiring pre-screening and, likely, reducing stigma for participants, it is important to note that our findings are likely not generalizable to targeted interventions, in which all group members meet a certain symptom threshold. Secondly, when conducting studies in schools (and other naturalistic settings), researchers often encounter less control over the setting. Consequently, several factors can pose a threat to internal validity, such as students sharing information about their group with other students and academic schedules requiring delays in the collection of data.

In conclusion, using an implementation partner to further scale up the Shamiri intervention is a promising way of expanding the reach of this effective school-based intervention.

## Trial status

Participant recruitment had not started before this manuscript was first submitted to Trials. Participant recruitment started on May 22, 2023. This manuscript was fist uploaded in its current form on May 18, 2023. Earlier submission was not possible because the research team was too busy preparing the intervention itself. Participant recruitment was completed on July 14, 2023.

### Supplementary Information

**Additional file 1.** Shamiri Wellness Program Group Leader Protocol.

**Additional file 2.** Emergency protocol.

**Additional file 3.** Sociodemographic questionnaire.

**Additional file 4.** Power calculation

## Data Availability

The article publication resulting from the present data will be available to the public. De-identified data that underlies results reported in the article will also be available upon request through open science foundation and will be available up to at least 3 years after the last follow-up.

## Abbreviations

TAU: Treatment as usual
FID: Fonds d’Innovation pour le Développement
WHO: World Health Organization
LMIC: Low- and middle-income countries
DALY: Disability-adjusted life years
SSA: Sub-Saharan Africa
AMHRFT: Africa Mental Health Research and Training Foundation
PHQ_8: 8-Items Patient Health Questionnaire
GAD- 7: 7-Item Generalized Anxiety Disorder Screener
MSPSS: Multidimensional Scale of Perceived Social Support
PCS: Perceived Control Scale
GQ_6: 6-Item Gratitude Questionnaire
ES: Effect sizes
MICE: Multivariate Imputation by Chained Equations
PI: Principal investigator
SWEMWBS: Short Warwick-Edinburg Measure of Mental Well-Being Scale
SES: School Engagement Scale
IRB: Institutional Review Boards
PACTR: Pan African Clinical Trials Registry
MoU: Memorandum of understanding
