# Supplementary material for: Testing pathways to scale: study protocol for a three-arm randomized controlled trial of a centralized and a decentralized (“Train the Trainers”) dissemination of a mental health program for Kenyan adolescents
Source: Trials. 2023 Aug 13;24:526. doi: 10.1186/s13063-023-07539-y (PMC10424401; doi:10.1186/s13063-023-07539-y)
Supplement: Supplementary file 2 — Additional file 2. Emergency protocol. [file 13063_2023_7539_MOESM2_ESM.docx]

**Emergency Protocol: Anansi study**

*Risk Guideline Literature Review:* This Emergency Protocol for participant risk scenarios (which can be flexibly used for all participants, but is especially designed for use with participants still in schools) was developed using the American Academy of Pediatrics guidelines for child and adolescent suicide risk, which encourage explicitly asking about suicidal thoughts, intent, plans, and history, as well as assessing risk and protective factors and triangulating interview findings with symptom measures (Shain, 2016). These guidelines also suggest sensitivity and caution and having a natural conversation about how the youth is doing, not just firing off a series of questions about suicide (Shain, 2016). Additionally, related guidelines from the American Academy of Child and Adolescent Psychiatry suggest balancing participant confidentiality and comfort with the need to inform guardians (i.e., teachers in the case of this present study) of child or adolescent risk.  In all cases in which harm may be imminent (i.e., high risk) it is necessary to inform the guardian of youth risk (Sondheimer, 2010; Shain, 2016), however, in cases in which the harm is not intended or imminent, steps are less clear-cut and must involve balancing participant comfort and confidentiality with safety, where safety is, of course, most important (Sondheimer, 2010). In an effort to achieve the right balance, we will inform school staff of students who our assessment identifies as posing medium or high risk of suicide; we will not *routinely* inform schools of low-risk cases, but our expert clinician will consider that step in low-risk cases if it seems appropriate. Of course, in all low-risk cases, we will continue monitoring and evaluation and inform the school if the student rises to a higher risk level.

*Study Procedures:* The study staff (other than the nurse, when judged appropriate by schools for the youth to be asked about IPV and drug use) will not systematically ask participants about risk of harm to self or others.  This is in order to avoid unnecessary stigma and discomfort.  However, if a participant displays potentially serious mental health concerns (e.g., if a participant mentions thoughts of/desire to harm others) or other potentially serious health and safety concerns (e.g., abuse, drug addiction), the study staff will be trained to further assess the situation (by asking, for example, about thoughts of harm, and, depending on their response, methods, plans, intent, and history, or about desire and ability to stop drug use). Then, they will be trained to immediately speak with a doctoral-level supervisor on the study team, if needed. The study staff (and in some cases supervisor) will then assist with triage and, if appropriate, follow-up and referral to appropriate local resources.

***Suicide/Self-harm Risk:***

For risk of harm to self or others, the possible levels of risk and actions taken for each level of risk are listed below:

1. No risk -- for those with **no thoughts of/risk of harm to self or others**. In these cases, no further monitoring or follow-up will be necessary.
2. Low-risk -- for those with **only thoughts of harm to self or others, and no history of attempt, and no specific methods, plans, or intent.** In these cases, study staff will continue to monitor this participant by contacting them again via phone or email, and will consult some combination of trained counseling psychologists in our team with at least a Master’s qualification (Brenda Ochuku and Veronica Ngatia) and doctoral qualification in counseling psychology, clinical psychology, or psychiatry (Dr. Christine Wasanga, Dr. Victoria Mutiso, Dr. Christine Musyimi, and Dr. David Ndetei). We will offer resources as recommended by these experts.
3. Medium risk -- for those with **thoughts of harm to self or others + any plans** (e.g., place, method, time, final preparations) **or history of attempt over six months prior.** Additionally, some facing risk of non-life-threatening or not ongoing abuse or using drugs may fall into this risk level. In these cases, study staff will continue to monitor this student through the intervention period, will consult with the PIs and will consult some combination of trained counseling psychologists in our team with at least a Master’s qualification (Brenda Ochuku and Veronica Ngatia) and doctoral qualification in counseling psychology, clinical psychology, or psychiatry (Dr. Christine Wasanga, Dr. Victoria Mutiso, Dr. Christine Musyimi, and Dr. David Ndetei). We will offer resources as recommended by these experts. Additionally, in these cases, the study staff will make every attempt to have the participant speak one-on-one with a trained counseling psychologist in our team, either in person or over the phone. The study team led by Dr. Wasanga will then determine the most appropriate course of action based on their expert clinical judgment. We may recommend measures including, but not limited to, confiscation of means, referral to community resources, consultation with school administrators, referral to psychiatrists or psychologists, and involvement of a trusted teacher, parent, or administrator.

1. High-risk -- for those with **thoughts + plans + intent or thoughts + recent (less than six months prior) history of attempt to seriously harm/kill themselves or someone else**. Additionally, some facing risk of life-threatening or severe ongoing abuse or using drugs particularly frequently or in a particularly risky fashion may fall into this risk level. In these cases, study staff will immediately consult with Dr. Christine Wasanga, a trained PhD-level counseling psychologist. Then, we will require that the participant speak one-on-one with Dr. Christine Wasanga or another doctoral-level supervisor with clinical training, either in person or over the phone. Dr. Wasanga and/or another doctoral-level supervisor will then determine the most appropriate course of action based on their expert clinical judgment. They may recommend measures including but not limited to: confiscation of means that could be used to harm self or others, consultation with school administrators, referral to psychiatrists or psychologists, referral to other community resources, and involvement of a trusted teacher, parent, or administrator. In cases of imminent threat to health and safety, the study team may call on local authorities and customs as needed to help ensure the health and safety of participants and staff. Professor John Weisz and doctoral-level affiliates of AMHRTF will also be available on call should need be.

1. In less severe and pressing circumstances, we may, after consultation with some or all professionals listed above refer these students to the appropriate school administrator (e.g., school chaplain, school principal) or community resource. In these cases, we will follow the standard referral procedures used by these schools and organizations. We will speak to school administration in cases of potential risk for which the study PI and other experts deem it necessary, and for cases in which the study PI is unsure about the need to consult the administration; we will contact the IRB to inquire about an appropriate path forward.

***Violence/Abuse Risk:***

The possible levels of risk and actions taken for each level of risk are listed below:

- No risk -- for those with no history of intimate partner violence.
  - In these cases, no further monitoring or follow-up will be necessary.

- Low-risk -- for those who answer “Yes” to either question 1 or question 6 on the intimate partner relationships scale and answer “No” to all other questions. In other words, **cases of verbal abuse and/or humiliation ONLY**.
  - In these cases, study staff will continue to monitor this student, will consult with Dr. Christine Wasanga, who is a trained counseling psychologist, or another supervisor with a clinical degree in clinical or counseling psychology or psychiatry, and will offer resources to the student as recommended by Dr. Wasanga.
  - Because the student will be monitored, if they do at some point reach medium or high risk, school staff will be notified, and the student will be very strongly encouraged to seek individual professional care.

- Medium risk -- those who have either been threatened or have threatened their violence however **have never inflicted harm or been a victim of any form of violence** will be classified as medium risk. This corresponds with question 2 and 7 as well as the first part of question 4 and 9 *intimate partner relationships* scale.
  - In these cases, study staff will immediately consult with Dr. Christine Wasanga, who is a trained counseling psychologist, or another supervisor with a clinical degree in clinical or counseling psychology or psychiatry, and will offer resources to the student as recommended be this expert.  Additionally, in these cases, the study staff will make every attempt to have the student speak one-on-one with Dr. Wasanga (or another expert), either in person or over the phone.  Dr. Wasanga or another expert will then determine the most appropriate course of action based on her expert clinical judgment.  She may recommend measures including, but not limited to, confiscation of means, consultation with school administrators, referral to psychiatrists or psychologists, and involvement of a trusted teacher, parent, or administrator.
  - If Dr. Wasanga or another expert deems a student to be at this risk level, they report the student risk to the school, which is the student’s current guardian.
  - At this risk level, we will also make every effort to encourage the student to have psychotherapy sessions with a local mental health professional.

- High-risk -- for **those who have experienced past or current physical or sexual violence** will be classified as high risk. Additionally, **those who have inflicted such violence** will also fall under this risk category. This corresponds with questions 3, 4, 5 as well as questions 8, 9, and 10 on the *intimate partner relationships* scale.
  - In these cases, study staff will immediately consult with Dr. Christine Wasanga or another supervisor with a terminal degree in clinical or counseling psychology or psychiatry.  Then, we will require that the student speak one-on-one with Dr. Christine Wasanga or another expert, either in person or over the phone.  This expert will then determine the most appropriate course of action based on her expert clinical judgment.  She may recommend measures including but not limited to: confiscation of means that could be used to harm self or others, consultation with school administrators, referral to psychiatrists or psychologists, and involvement of a trusted teacher, parent, or administrator. In cases of imminent threat to health and safety, the study team may call on local authorities and customs as needed to help ensure the health and safety of participants and staff.
  - If Dr. Wasanga or another expert deems a student to be at the high-risk level, they will report the student risk to the school, which is the student’s current guardian.
  - At this risk level, we will also make every effort to encourage the student to have psychotherapy sessions with a local mental health professional.

**Substance Abuse Risk:**

The possible levels of risk and actions taken for each level of risk are listed below:

- No risk -- for those with no previous or current substance use.
  - In these cases, no further monitoring or follow-up will be necessary.

- Low-risk – for **those who are using a substance(s) however very infrequently (i.e. once or twice a year**). A total score of 1 to 7 on the AUDIT and/or a “once or twice” response to question 2 of the ASSIST indicates a low risk.
  - In these cases, study staff will continue to monitor this student, will consult with Dr. Christine Wasanga, who is a trained counseling psychologist, or another supervisor with a clinical degree in clinical or counseling psychology or psychiatry, and will offer resources to the student as recommended by Dr. Wasanga.
  - Because the student will be monitored, if they do at some point reach medium or high risk, school staff will be notified, and the student will be very strongly encouraged to seek individual professional care.

- Medium risk -- for **those who are using a substance(s) often (i.e. once or twice a month) OR for those who have previously used a substance(s) more frequently than once or twice a month, however not in the past 6 months**. Scores from 8 to 14 on the AUDIT and/or a “Monthly” response to question 2 of the ASSIST indicate a medium risk.
  - In these cases, study staff will continue to monitor this student will consult with Dr. Christine Wasanga, who is a trained counseling psychologist, or another supervisor with a clinical degree in clinical or counseling psychology or psychiatry, and will offer resources to the student as recommended be this expert.  Additionally, in these cases, the study staff will make every attempt to have the student speak one-on-one with Dr. Wasanga (or another expert), either in person or over the phone.  Dr. Wasanga or another expert will then determine the most appropriate course of action based on her expert clinical judgment.  She may recommend measures including, but not limited to, confiscation of means, consultation with school administrators, referral to psychiatrists or psychologists, and involvement of a trusted teacher, parent, or administrator.
  - If Dr. Wasanga or another expert deems a student to be at this risk level, they report the student risk to the school, which is the student’s current guardian.
  - At this risk level, we will also make every effort to encourage the student to have psychotherapy sessions with a local mental health professional.
- High-risk -- for **those who are using a substance(s) daily or almost daily**. A score of 15 or more on the AUDIT and/or a “weekly” or “daily” response to question 2 of the ASSIST will be classified as high risk.
  - In these cases, study staff will immediately consult with Dr. Christine Wasanga or another supervisor with a terminal degree in clinical or counseling psychology or psychiatry.  Then, we will require that the student speak one-on-one with Dr. Christine Wasanga or another expert, either in person or over the phone.  This expert will then determine the most appropriate course of action based on her expert clinical judgment.  She may recommend measures including but not limited to: confiscation of means that could be used to harm self or others, consultation with school administrators, referral to psychiatrists or psychologists, and involvement of a trusted teacher, parent, or administrator. In cases of imminent threat to health and safety, the study team may call on local authorities and customs as needed to help ensure the health and safety of participants and staff.
  - If Dr. Wasanga or another expert deems a student to be at the high-risk level, they will report the student risk to the school, which is the student’s current guardian.
  - At this risk level, we will also make every effort to encourage the student to have psychotherapy sessions with a local mental health professional.

Professor John Weisz and Professor Eve Puffer will also be available on call should need be.

If less urgent pressing problems are uncovered in the risk assessment, we may, after consultation with Dr. Wasanga (a counseling psychologist) and Dr. Weisz (a clinical psychologist) or other experts, refer these students to the appropriate school administrator (e.g., school chaplain, school counselor, school principal). In these cases, we will follow the standard referral procedures used by these schools. For cases in which the study PI is unsure about the need to consult the administration or is unsure about other potential steps, we will contact the Kenyatta University IRB to inquire about an appropriate path forward.
