# Supplementary material for: Testing pathways to scale: study protocol for a three-arm randomized controlled trial of a centralized and a decentralized (“Train the Trainers”) dissemination of a mental health program for Kenyan adolescents
Source: Trials. 2023 Aug 13;24:526. doi: 10.1186/s13063-023-07539-y (PMC10424401; doi:10.1186/s13063-023-07539-y)
Supplement: Supplementary file 3 — Additional file 3. Sociodemographic questionnaire. [file 13063_2023_7539_MOESM3_ESM.docx]

### Demographics Questionnaire

**Age** ______________

**Gender**

Female

Male

Prefer not to answer.

Other

If other, please specify ________________________

**Form**

Form 1

Form 2

Form 3

Form 4

**Religion/Spirituality**

Christian catholic

Christian protestant

Muslim

Buddhist

Traditional African

No religion

Other? Please specify________________________

**At home, how many parents do you live with? (Please cross one)**

Both Parents

Single parent

No parents

**What county do you live in while away from school?**

**Have any of your parents passed away? (Please cross one)**

Father

Mother

Both

None

**What is your father’s highest level of education? (Please cross one)**

University

Secondary School

Primary School

Not aware

**What is your mother’s highest level of education? (Please cross one)**

University

Secondary School

Primary School

Not aware

**Are you involved in co-curricular activities like clubs and societies? (Please cross one)**

Extremely involved

Quite involved

Not involved at all

**Are you part of a school sports team, like basketball or rugby? (Please cross one)**

Yes

No

**How would you rate your academic performance? (Please cross one)**

Excellent

Very good

Good

Satisfactory

Not satisfactory
