# Supplementary material for: Testing pathways to scale: study protocol for a three-arm randomized controlled trial of a centralized and a decentralized (“Train the Trainers”) dissemination of a mental health program for Kenyan adolescents
Source: Trials. 2023 Aug 13;24:526. doi: 10.1186/s13063-023-07539-y (PMC10424401; doi:10.1186/s13063-023-07539-y)
Supplement: Supplementary file 4 — Additional file 4. Power calculation [file 13063_2023_7539_MOESM4_ESM.pdf]

# Power Analysis and Sample Size Determination for the “Anansi Project”

**Thomas Rusch**
WU Vienna

**Abstract**

This report provides power analyses and sample size determinations for the Anansi project. I document assumptions behind the analysis, the models and outcomes as well as the results. In summary: To achieve a power of at least 80% for effect sizes as observed in the JAMA study over both GAD and PHQ at any of the time point for which we had practically relevant effect sizes ($d > 0.2$) in the JAMA study, the estimated necessary minimum sample size was $n^* = 1554$ (including an attrition assumption of 40% in the worst case). This is the minimum sample size for detecting an effect with 80% power that is at least as big as the one at `MP` for PHQ, corresponding to a $d = 0.22$. For this $n^*$ the point estimate for the power at this time point was 82.4% with a 95%-CI of 80% to 85%. Other scenarios are also documented in the following.

*Keywords*: Shamiri, Anansi, multilevel models, power analysis, sample size.

## 1. Introduction

Here we document the power analysis and sample size determination (on level-2, the level of participants) for the Anansi study. Overall this is a difficult undertaking as we need to consider:

- Complicated nesting structure which we must tackle with multilevel models.
- Attrition in the data that is only partially matched by the data.
- Two outcome measures (PHQ and GAD) that do not behave the same but we need to have only one sample.
- Different effects and attrition at different time points but we need one longitudinal sample.

We do sample size calculations under a sort of bad-case scenario. We want to make sure we achieve at least a target power of $P\%$ under our assumptions. Therefore we aim for the minimum sample size we need to get under a non-optimal but realistic scenario (attrition, effect sizes, etc.); this estimate will therefore be conservative. In practice and if the situation is better than assumed, a smaller sample size might work just as well (say, if attrition is less, if effect sizes are larger, if noise is lower in reality, etc) but there’s no guarantee this better situation will happen. So in a way what we do here is risk management—we hedge against

realistic but suboptimal outcomes in a study by making sure we have a sample size large enough. If things get even worse than we expect of course we'd be too optimistic.

Notationwise, I'll distinguish between the effective sample size ($n_{eff}$) that is the sample size returned by the simulations and the necessary minimal sample size to achieve the effective sample size given the attrition asumptions, $n^* = a * n_{eff}$, with $a$ an attrition correction factor described below. The power numbers given always correspond to an effective sample size (or the corresponding $n^*$). We need to use the $n^*$ to know how many participants we need *at least*.

## 2. Summary

In Table 1 I list the necessary minimal sample sizes $n^*$, effective sample sizes $n_{eff}$, the standardized $d$ and unstandardized $b$ effect sizes for which we expect at least a power of 80% over all time points and both dependent variables under the assumptions/setup discussed below. I also list the dependent variable associated with that $n^*$; we always just need to take the smallest standardized effect over both DV into account to get the minimal necessary sample size for a given power because for the DV with the larger effect the power will be higher for this sample size anyway. We also list the 95% confidence interval (CI) of the estimated power at that scenario and which time point it was (if there will be the decision to be made to not do it over all time points but just for, say, `EP`).

| $n^*$ | $n_{eff}$ | $d$ | $b$ | 95%-CI for Power | DV | Time |
|---|---|---|---|---|---|---|
| 1554 | 1400 | 0.22 | 0.89 | $(0.8, 0.85)$ | PHQ | MP |
| 666 | 600 | 0.3 | $-1.24$ | $(77.28, 84.35)$ | PHQ | EP |
| 1110 | 1000 | 0.25 | $-1$ | $(77.07, 84.16)$ | PHQ | EP |
| 1443 | 1300 | 0.25 | $-1$ | $(78.13, 85.09)$ | PHQ | FU |
| 1221 | 1100 | 0.28 | $-1.05$ | $(80.21, 85.00)$ | GAD | FU |
| 1221 | 1100 | 0.25 | $-0.95$ | $(76.22, 83.42)$ | GAD | EP |

Table 1: Minimal sample size $n^*$, effective sample size $n_{eff}$, unstandardized effect size $b$ and standardized effect size $d$ for which a power of at least 0.8 is achieved at any time point (over both dependent variables). The dependent variable that gave rise to that sample size is given in the column DV and the time point at which this happened is given in column Time.

The table is to be read like this: **Use** $n^* = 1554$ **to make sure that we have at least** 80% **power over all the assumed effects over all three time points for both DV (lowest** $d$ **overall)**. This is the information we're after and my recommendation.

But we may want to be more flexible and decide we no longer care about an effect size at `MP`; then this table lists other $n^*$ that may be of interest at which point we'd have around 80% power for other effect sizes. For example, say we decide to only care about `EP`, then for this assumed effect size a $n^* = 666$ will suffice for both DV (the power at the other time points with that sample size is reported below). If we care about a $d = 0.25$ over any time point and any DV we would use $n^* = 1443$; if we'd decide to only care about GAD (not PHQ) then $n^* = 1221$ would suffice for a $d = 0.25$. If we'd care about an assumed unstandardized effect of $b = -1$ over any time point for PHQ only we'd use $n^* = 1443$ (the one for `FU`) but if we only cared about $-1$ at `EP`, $n^* = 1110$ would suffice.

# 3. Assumptions and Status Quo

Here we document the assumptions that we make for the sample size determination and power analysis as well as discuss the status quo.

## 3.1. Status Quo

The design that is planned is that we have students (participants or kids, `Participant_ID`) from different counties and schools randomly assigned to a control (TAU) or a treatment condition (Factor `Condition`). The treatment condition gets the Shamiri intervention. We measure students symptoms with the PHQ-8 and the GAD-7 over the course of 8 weeks (factor `Time`): Baseline (`BL`, 0 weeks in), midpoint (`MP`, 2 weeks in), end point of the intervention (`EP`, 4 weeks in) and at 1 Month follow-up (`FU`, 8 weeks in). We're specifically interested in the power of tests of significance between the treatment condition and the control condition (called TAU) at all time point other than `BL` for different sample sizes and the minimum sample size that is estimated to give us a specific power.

There will be two implementers: Shamiri Institute (SHA) and AMHRTF (AMH). In this analysis we'll check power/sample size of TAU vs. treatment and *pretend that the effects of SHA and AMHRTF are the same.* This will give one power analysis/sample size determination.

We have a pre-study that we use as our blueprint: The JAMA Psychiatry study with data from 2019. The nesting structure is assumed to be the same, so there are repeated observations per student that are nested in students. Students are nested in administration groups (where all got the intervention or the control; factor `Group`) which are nested in nested within their group leaders (`Group_Leader`). The `groups` are also nested within schools (so no administrative group consists of students from different schools; factor `School`). The `Group_Leader` is crossed with school (as each leader could have groups in more than one school).

We have the control variables `Age`, `Gender` and `Form`. As dependent variables (`DV`) we'll use the sum score of PHQ and GAD respectively.

Based on that we have the following model we fit (in `lmer` pseudo code):

```
> DV ~  Age + Gender + Form + Time * Condition
>       + (1|Participant_ID:Group:Group_Leader)+ (1|Group:Group_Leader)+ (1|Group_Leader) + (1|School)
```

Since we're interested in the power of tests of significance between the control condition (called TAU), and the treatment condition (SH) we are interested in mean(ConditionSH)-mean(ConditionTAU) at `MP`, `EP` and at `FU`. This is easiest to determine by reparametrizing the model so that `EP`, `MP` and `FU` are reference levels in turn and just look at the t-test of `ConditionSH=0` (assuming the reference for Condition is `TAU`) because we have mean(conditionSH) = `Intercept+ConditionSH` and mean(conditionSH)=`Intercept`. Otherwise it would mean looking at `ConditionSH=0` at reference level (say `EP`) and at `ConditionSH+ConditionSH:TimeFU=0` as mean(conditionSH,FU)=`Intercept+TimeFU` and mean(conditionSH,FU)=`Intercept+ConditionSH+TimeFU+ConditionSH:TimeFU` and we'd need to look at a generalized linear hypothesis test for that effect. For `MP` that would be equivalent just swap the `TimeFU` for `TimeMP`.

Since we must do this with multilevel models, there are no closed-form solutions and we have to simulate. I'll use the R package **simr** for this.

### 3.2. Assumptions and Parameters

Power/sample size determination for these models entails making many assumptions about hypothesized effect sizes and other parameters. I'll use values we obtained in the previous studies *without imputation*. Note we don't have a 1 month follow-up in the 2019 study, so I equate the 2 week follow-up in the JAMA study in 2019 with the 1 month follow-up (so we equate `FU` in Anansi with `2WFU` in 2019).

Note that due to our having to test fixed effects, we must not use the REML criterion for estimation in mixed-effect models, so the effects (mainly random effects) will differ a bit between what I have here and what is in the ShamiriDASupplement (where we used REML). That shouldn't make a big difference for the sample size/power for testing fixed effects.

For PHQ (based on FML not REML):

- Fixed effects: We expect the average values of DV at `EP` in the treatment group to be 6.4 for the reference kids (Gender=male, Form=1, Age=min(age)) (`Intercept`). Then for the reference kids in the control group at `EP` we expect a mean of 7.64, so the average score to be higher by 1.24 in control, so $mean(treatment) - mean(control) = b = -1.24$. At 1 month follow-up (`FU`) we expect a shift down for the treatment group of $-1.03$ to a mean of 5.37. For the control at `FU` we expect a decrease from `EP` to `FU` of $-1.13$ ($-1.03-0.1$) to a mean of 6.51. This makes the difference between the treatment and control group at `FU` to be $b = -1.13$ (treatment-control). We thus calculate power/sample size with a hypothesized effect size between control and treatment of $b = -1.24$ at `EP` and of $b = -1.13$ at `FU`. At `MP` the difference of the means of treatment and control is $b = 0.89$ (so control did better than treatment). These unstandardized effect sizes roughly translate to $d = 0.29$ at `EP`, $d = 0.27$ at `FU` and $d = 0.22$ at `MP` respectively when standardizing them by dividing with the residual standard error. All other fixed effects are assumed to be as in the JAMA study.

- Random effects: The residual standard error we expect to be 4.09. The standard deviation of the random effects we expect to be 2.32 for the intercept of `Participant_ID`, 0.28 for the intercept of `Group_Leader` and 0.45 for the intercept of `School`. For `Group` it is 0 (or ignored).

- Attrition: We expect 30% attrition at midpoint and endpoint (individually, not cumulative) and 40% at follow-up (individually, no cumulative), so this needs to be a corrective factor. This is extremely high attrition.

For GAD (based on FML not REML):

- Fixed effects: We expect the average values of DV at `EP` in the treatment group to be 6.6 for the reference kids (Gender=male, Form=1, Age=min(age)). Then for the reference kids in the control group at `EP` we expect the average score to be higher by $b = 1.68$ (mean of 8.28). At 1 month follow-up (`FU`) we expect a further shift down for the treatment group of $-1.25$ (to a mean of 5.35). For the control at `FU` we expect a decrease from `EP` to `FU` of $-1.25 - 0.61 = -1.84$ (to a mean of 6.45), which means a difference in means of $b = -1.68 - 0.61 = -1.07$ between treatment and control. So we calculate power/sample size with an hypothesized effect size between control and treatment ($mean(treatment) - mean(control)$) of $b = -1.68$ at `EP` and of $b = -1.07$

at FU. These unstandardized effect sizes roughly translate to $d = 0.44$ and $d = 0.28$ respectively when standardizing them by dividing with the residual standard error. All other fixed effects are assumed to be exactly as in the JAMA study. At MP the difference in the means between treatment and control is so close to zero as to not be practically relevant ($d = 0.15$) and therefore ignored subsequently.

- Random effects: The residual standard error we expect to be 3.8. The standard deviation of the random effects we expect to be 2.1 for the intercept of `Participant_ID`, 0.4 for the intercept of `Group` and 0.0005 for the intercept of `School`. For `Group_Leader` it is 0.3.
- Attrition: We expect 30% attrition at midpoint and endpoint (individually, not cumulative) and 40% at follow-up (individually, no cumulative), so this needs to be a corrective factor. This is extremely high attrition.

Note that from EP to FU we assume further improvement in symptoms as this is how it presents itself in the previous data. For testing the effect between control and treatment at each time point, it doesn't really matter what the pattern (down or up) from EP to FU is as long as the pattern would be seen in both groups.

Test parameters:

- alpha: 0.045 (the correction of Baayen et al. (2008))
- power: 0.8

# 4. Sample Size Determination and Power Analysis

## 4.1. Preliminary Comments

I'll calculate the needed sample size $n^*$ for a power of 0.8 with an type-I-error rate *per test* of 0.045 (a correction suggested by Baayen et al. (2008) for the t-statistic). We have at most 3 tests of interest, one for ($treatment - control$) at MP, EP and one for FU. This is not adjusted to the familywise error rate, so overall we have a higher chance for a type-I-error on all three tests combined (around 0.13). This means we're a bit too liberal on each of the individual in the sense of getting more power/less sample size then we would if accounted for the familywise error.

We use the data from the JAMA study in 2019 as the blueprint. Overall we had 413 participants in that study. We didn't have a 1 month follow-up (FU) as is planned in Anansi, but a 2 week follow-up (2WFU). I'll hypothesize that what was observed 2WFU in 2019 is also what we'll observe at the FU in Anansi.

I've been instructed that we want to determine the necessary minimum sample size for all differences between Control and Treatment condition at all the time points MP and EP and FU. Note we don't want this for BL because we randomize at BL; we don't want to have significant differences in terms of the outcome before we start with a treatment because then the randomization wouldn't have worked.

Having tests at three time points leads to the following observation: Let us for the moment assume there is no attrition. Then we will observe the same participants at each time point as it is a longitudinal study. This means the necessary sample size for differences in condition at different time points cannot be changed (the participants with intent to be treated need to be recruited beforehand and stay the same). This means that we cannot have different sample sizes at different time points (again, if we had no attrition). This is important because this implies we only need to find the necessary sample size to detect the smallest difference between treatment and control condition that we observed over any of the time points we're interested in `MP`, `EP` and `2WFU`.

Since both DV will be used for the same students, we need to select the maximum number of students per timepoint over both DV to get the overall sample size $n^*$.

Note since this is a simulation, the concrete values can vary a bit between simulations. I'll set a seed for reproducibility. If you need to make sure the estimated power is conservative, then one can use the low boundary of the CI as the associated power (and perhaps a higher sample size so that the lower bound of the CI exceeds the target power of 80%).

Due to how the software we're using actually works, we have to remove missing data prior to running the simulations. This means that the effects estimated on the data without missings would differ slightly because there are some participants for which we lose all the observations; in the model in 2019 they don't need to be removed and therefore have an impact due to the partial pooling effect of mixed models. To accomodate that I'll use the object we got from removing the missings and add to it the fixed effects and random effects statistics from the 2019 study; this makes the whole thing doable and luckily doesn't effect estimates in terms of the standardized effect sizes at `MP`.

Removing the missings prior to the simulations means that we carry the attrition at each time point with us automatically (the software can't deal with `NA`s). Internally in the simulations the number of participants gets replicated randomly (upscaled) to achieve the final sample size for simulations. This means the number of missings of each participant will be upscaled as well. This is the effect: due to the original participant number being 405 observations (after removal of complete missings) we potentially have 405 observations per time point. Since we have 5 time points in the data, that means potentially 2025 level-1 observations (measurements per person). Note that in the JAMA data we actually have 408 kids and thus potentially 2065, but 3 kids drop out completely after removal of missings so we'll work without these 3. Now, say in our simulations we want to upscale to 2000 people instead of 405; of the 405 people we replicate as many people as we need to now have 2000 and add them to the data set. But not all people had full records on all time points, so we do not have $2000 * 5 = 10000$ level-1 observations, because depending on which person gets replicated, we also replicate the missing values of that person. So if we had an average missing values percentage of 15% over all people in the data, then upscaling to 2000 students effectively means upscaling to $10000 - (10000 * 0.15) = 8500$ level-1 observations instead of 10000. This is how the attrition/missings in the JAMA study carries over into the simulation. Unfortunately, we cannot fully control in the software how much attrition is carried over in the simulation at each time point to match exactly the observed attrition of each time point; but due to the random replication of people it should vary only slightly around the average, which is about 30% overall (1431 level-1 observations as compared to 2025 if we had full records of everyone). Therefore with respect to sample size determination, the number of participants obtained via the simulation only needs to be corrected by the excess number of attrition that we assume

as compared to what is carried through the simulation.

We assume an attrition from BL to EP of 40% and of 30% from BL to FU. The attrition carried over into the simulation is around 30% both for PHQ and GAD. For both PHQ and GAD, this is therefore an excess attrition of 0% from BL to MP, 10% percentage points from BL to EP and 0% percentage points from BL to FU to the attrition already included in the model from 2019 that was carried through into the simulations (which was 30% on average). Thus we have to scale up the effective minimal sample size $n_{eff}$ that we obtained in the simulation with a factor of $a = 1/(1 - 0.10) = 1.11$ to match the highest expected attrition of 40% EP and ensuring that the power obtained for $n_{eff}$ is met by using $n^* = a * n_{eff}$. For FU $a = 1$.

Overall therefore since we use the same sample of participants for GAD and PHQ, we just need to multiply whatever minimal sample size we get in the simulations under 2019 attrition with $a = 1.11$ as $1.11 * n_{eff} > 1 * n_{eff}$ to get a sample size that will have at least $n_eff$ and therefore the target power with the attrition as assumed (same argument as before with the effect sizes). Note that this is again conservative because if attrition happens to be less, then that sample size will suffice (we will have more power then).

For the test, I'll use the t-statistic with the Satterthwaite ddf calculation (it is not yet clear in the literature which ddf should be used in general). At this level of observations for participants, the asymptotics should be ok and we shouldn't get too much divergence between different ddf calculations though (I usually prefer Kenward-Roger but we'd have to set up the model differently so as to isolate the effect instead of using Timef*Condition and this is much easier with Satterthwaite). As mentioned before I use $\alpha = 0.045$ instead of 0.05 as Baayen et al. (2008) suggest.

## 4.2. PHQ

For PHQ we observed significant differences between Wellness (the Shamiri treatment condition) and Study-Skills (the control condition) at EP. We did not find a significant effect at MP. We did not find significant effects at 2WFU (but barely). These results are repeated subsequently with REML=FALSE. The fitted model was (now with REML=FALSE).

```
> ## full specification of the nesting as I think it should be - explicit
> phqModel_2b <- lmer(PHQsum ~ Timef * Condition + Gender + Form +
+                        (1|Group_Leader) + (1|Group:Group_Leader) +
+                        (1|Participant_ID:Group:Group_Leader) + (1|School),
+                     data=dfss2, REML = FALSE, verbose=0)
> summary(phqModel_2b)
```

```
Linear mixed model fit by maximum likelihood . t-tests use Satterthwaite's
  method [lmerModLmerTest]
Formula: PHQsum ~ Timef * Condition + Gender + Form + (1 | Group_Leader) +
    (1 | Group:Group_Leader) + (1 | Participant_ID:Group:Group_Leader) +
    (1 | School)
   Data: dfss2

     AIC      BIC   logLik deviance df.resid
  8513.6   8613.9  -4237.8   8475.6     1425

Scaled residuals:
    Min      1Q  Median      3Q     Max
-2.5360 -0.6499 -0.0790  0.5834  3.6642

Random effects:
 Groups                             Name        Variance Std.Dev.
 Participant_ID:Group:Group_Leader (Intercept)  5.38628 2.3208
 Group:Group_Leader                 (Intercept)  0.00000 0.0000
 Group_Leader                       (Intercept)  0.07694 0.2774
 School                             (Intercept)  0.20361 0.4512
```

```
 Residual                                 16.72656 4.0898
Number of obs: 1444, groups:
Participant_ID:Group:Group_Leader, 408; Group:Group_Leader, 45; Group_Leader, 15; School, 4

Fixed effects:
                                  Estimate Std. Error         df t value
(Intercept)                        6.21170    0.60319   13.40408  10.298
TimefBL                            5.90304    0.45314 1121.56220  13.027
TimefMP                            2.86819    0.48873 1113.51434   5.869
Timef2WFU                         -1.02427    0.52530 1147.90592  -1.950
Timef7MFU                          0.69861    0.52533 1172.14621   1.330
ConditionStudy-skills              1.23768    0.54516  781.78925   2.270
GenderFemale                       0.94860    0.54058    7.04492   1.755
Form2                              0.09234    0.37570  382.16200   0.246
Form3                              0.98560    0.46359  416.27824   2.126
Form4                             -0.28864    0.80825  258.33220  -0.357
TimefBL:ConditionStudy-skills     -1.64943    0.63720 1124.42098  -2.589
TimefMP:ConditionStudy-skills     -2.12900    0.67818 1107.82999  -3.139
Timef2WFU:ConditionStudy-skills   -0.10820    0.73481 1148.49531  -0.147
Timef7MFU:ConditionStudy-skills    1.03652    0.75059 1164.39033   1.381
                                Pr(>|t|)
(Intercept)                     9.68e-08 ***
TimefBL                          < 2e-16 ***
TimefMP                         5.79e-09 ***
Timef2WFU                        0.05143 .
Timef7MFU                        0.18383
ConditionStudy-skills            0.02346 *
GenderFemale                     0.12246
Form2                            0.80598
Form3                            0.03409 *
Form4                            0.72130
TimefBL:ConditionStudy-skills    0.00976 **
TimefMP:ConditionStudy-skills    0.00174 **
Timef2WFU:ConditionStudy-skills  0.88296
Timef7MFU:ConditionStudy-skills  0.16756
---
Signif. codes:  0 âĂŸ***âĂŹ 0.001 âĂŸ**âĂŹ 0.01 âĂŸ*âĂŹ 0.05 âĂŸ.âĂŹ 0.1 âĂŸ âĂŹ 1
optimizer (nloptwrap) convergence code: 0 (OK)
boundary (singular) fit: see help('isSingular')
```

and the condition differences at each time point were

```
> lsm2 <- ls_means(phqModel_2b,which="Timef:Condition",pairwise=TRUE)
> lsm2[c("TimefMP:ConditionWellness - TimefMP:ConditionStudy-skills",
+        "TimefEP:ConditionWellness - TimefEP:ConditionStudy-skills",
+        "Timef2WFU:ConditionWellness - Timef2WFU:ConditionStudy-skills"),]
```

```
Least Squares Means table:

                                                               Estimate
TimefMP:ConditionWellness - TimefMP:ConditionStudy-skills      0.891317
TimefEP:ConditionWellness - TimefEP:ConditionStudy-skills     -1.237678
Timef2WFU:ConditionWellness - Timef2WFU:ConditionStudy-skills -1.129475
                                                              Std. Error     df
TimefMP:ConditionWellness - TimefMP:ConditionStudy-skills       0.544057  766.3
TimefEP:ConditionWellness - TimefEP:ConditionStudy-skills       0.545157  781.8
Timef2WFU:ConditionWellness - Timef2WFU:ConditionStudy-skills   0.610009 1001.1
                                                              t value     lower
TimefMP:ConditionWellness - TimefMP:ConditionStudy-skills      1.6383 -0.176702
TimefEP:ConditionWellness - TimefEP:ConditionStudy-skills     -2.2703 -2.307824
Timef2WFU:ConditionWellness - Timef2WFU:ConditionStudy-skills -1.8516 -2.326518
                                                                  upper
TimefMP:ConditionWellness - TimefMP:ConditionStudy-skills      1.959336
TimefEP:ConditionWellness - TimefEP:ConditionStudy-skills     -0.167533
Timef2WFU:ConditionWellness - Timef2WFU:ConditionStudy-skills  0.067567
                                                              Pr(>|t|)
TimefMP:ConditionWellness - TimefMP:ConditionStudy-skills      0.10177
TimefEP:ConditionWellness - TimefEP:ConditionStudy-skills      0.02346 *
Timef2WFU:ConditionWellness - Timef2WFU:ConditionStudy-skills  0.06438 .
---
Signif. codes:  0 âĂŸ***âĂŹ 0.001 âĂŸ**âĂŹ 0.01 âĂŸ*âĂŹ 0.05 âĂŸ.âĂŹ 0.1 âĂŸ âĂŹ 1

  Confidence level: 95%
  Degrees of freedom method: Satterthwaite
```

That smallest effect is the effect of $b = 0.89$ at `MP` (see above). This roughly corresponds to a standardized effect size of $d = 0.22$ (dividing the effect by the residual standard error from the model above). There was also an interest in a standardized effect size of $d = 0.3$. I'll be running the simulation with $d = 0.3$ as well which is the effect size that corresponds to the

effect at EP (since that one is larger we will have less power for the sample size derived that way for the test at MP). Note that at MP the control did better than the treatment (so lower symptom mean for Study-skills and therefore the difference TimefMP:ConditionWellness - TimefMP:ConditionStudy-skills is positive).

It is also a good idea to do both effect sizes as the $d = 0.22$ is about 3/4 of the $d = 0.3$ so we hedge against a less optimistic outcome. In a nutshell therefore: The sample size determined with $b = 0.89$ will be the minimum sample size we need to detect *any* of our observed effects at MP, EP or FU in 2019.

Note that the random effect variance for Group was 0, so we remove it from the model. Here's the code to get the power analysis/ sample size determination:

```
> set.seed(666) #seed for reproducibility
> #re-parametrize so the effect at ConditionStudy-skills is directly the effect we want to detect.
> dfss2$Timef <- relevel(dfss2$Timef,ref="MP")
> dfss3 <- na.omit(dfss2) #remove the missings as otherwise simr::powerSim will only return errors
> ## original Model refitted
> phqModel_2b <- lmer(PHQsum ~ Timef * Condition + Gender + Form +
+                               (1|Group_Leader) +
+                               (1|Participant_ID:Group:Group_Leader) + (1|School),
+                               data=dfss2, REML = FALSE)
> #model object to use for simr (without missings)
> phq4simr    <- lmer(PHQsum ~ Timef * Condition + Gender + Form +
+                               (1|Group_Leader) +
+                               (1|Participant_ID:Group:Group_Leader) + (1|School),
+                               data=dfss3, REML=FALSE)
> ## Changing the values in phq4simr to how they were in the JAMA data set with missings
> fixef(phq4simr) <- fixef(phqModel_2b)
> sigma(phq4simr) <- sigma(phqModel_2b)
> VarCorr(phq4simr) <- VarCorr(phqModel_2b)
> ## Number of simulated data sets
> nsim<-1000
> #### Observed Power
> ## simulate observed power for the 2019 study at MP
> phq.ps1<-powerSim(phq4simr,test=fixed("ConditionStudy-skills",method="t"),
+                   nsim=nsim,alpha=0.045)
> phq.ps1  #observed power for the 2019 study for the efect at MP:
>          #We had a power of 34% (obviously low because we had a non-significant value)
>
>
> #### Power Curve Calculation
> ## Create more participants up to a maximum of 2000 that we then cut back (this is how simr works)
> phqmodelext <- extend(phq4simr, along="Participant_ID", n=2000) #we extend along the grouping variable
>                                         #Participant_ID, so we now have 2000 Participants instead of 405
>                                         #as in the 2019 study
> phq.ps2<-powerSim(phqmodelext,test=fixed("ConditionStudy-skills",method="t"),
+                   nsim=nsim,alpha=0.045) #93% power
>                                         #for 2000 participants
> phq.ps2 #we have a power of around 93% for 2000 participants (with replication of the attrition)
> ## Power curve. Where we cross the 80% threshod is where we have determined the necessary sample size
> phq.pc1 <- powerCurve(phqmodelext,test=fixed("ConditionStudy-skills",method="t"),
+                        along="Participant_ID", nsim=nsim,breaks=c(seq(500,1900,by=100)),
+                        alpha=0.045)
> phq.pc1
```

```
Power for predictor 'ConditionStudy-skills', (95% confidence interval),
by number of levels in Participant_ID:
```

```
   500: 42.70% (39.61, 45.83) - 1736 rows
   600: 48.30% (45.16, 51.45) - 2084 rows
   700: 54.40% (51.25, 57.52) - 2463 rows
   800: 58.80% (55.68, 61.87) - 2747 rows
   900: 64.80% (61.75, 67.76) - 3112 rows
  1000: 67.60% (64.60, 70.50) - 3453 rows
  1100: 72.90% (70.03, 75.63) - 3837 rows
  1200: 75.40% (72.61, 78.04) - 4125 rows
  1300: 79.50% (76.86, 81.96) - 4482 rows
  1400: 82.40% (79.90, 84.71) - 4822 rows
  1500: 85.70% (83.38, 87.81) - 5208 rows
  1600: 87.00% (84.76, 89.02) - 5500 rows
  1700: 89.80% (87.76, 91.61) - 5855 rows
  1800: 91.40% (89.49, 93.06) - 6197 rows
  1900: 92.40% (90.58, 93.97) - 6577 rows

Time elapsed: 3 h 20 m 36 s
```

We cross the 80% power boundary for an effect of at least $b = 0.89$ ($d = 0.22$) with around $n_{eff} = 1400$ participants. With the attrition correction factor of 1.11 this is $n^* = 1554$.

What power would we have for the test of the hypothesized values at `EP` and `FU`? (we use 1400 as that is the effective sample size)

```
> set.seed(666)
> ## FOR EP
> dfss2$Timef <- relevel(dfss2$Timef,ref="EP")
> dfss3 <- na.omit(dfss2) #remove the missings
> ## original Model refitted
> phqModel_2b <- lmer(PHQsum ~ Timef * Condition + Gender + Form +
+                              (1|Group_Leader) +
+                              (1|Participant_ID:Group:Group_Leader) + (1|School),
+                              data=dfss2, REML = FALSE)
> #model object to use for simr (without missings)
> phq4simr    <- lmer(PHQsum ~ Timef * Condition + Gender + Form +
+                              (1|Group_Leader) +
+                              (1|Participant_ID:Group:Group_Leader) + (1|School),
+                              data=dfss3, REML=FALSE)
> ## Changing the values in phq4simr to how they were in the data set with missings
> fixef(phq4simr) <- fixef(phqModel_2b)
> sigma(phq4simr) <- sigma(phqModel_2b)
> VarCorr(phq4simr) <- VarCorr(phqModel_2b)
> phqmodelext.1400 <- extend(phq4simr, along="Participant_ID", n=1400) #we extend along the grouping
>                                         #variable Participant_ID, so we now have 1400 groups = Participants
> phq.ps3<-powerSim(phqmodelext.1400,test=fixed("ConditionStudy-skills",method="t"),
+                   nsim=nsim,alpha=0.045)
> phq.ps3 #we have a power of around 98% for 1400 participants
> ## FOR FU
> dfss2$Timef <- relevel(dfss2$Timef,ref="2WFU")
> dfss3 <- na.omit(dfss2) #remove the missings
> ## original Model refitted
> phqModel_2b <- lmer(PHQsum ~ Timef * Condition + Gender + Form +
+                              (1|Group_Leader) +
+                              (1|Participant_ID:Group:Group_Leader) + (1|School),
+                              data=dfss2, REML = FALSE)
> #model object to use for simr (without missings)
> phq4simr    <- lmer(PHQsum ~ Timef * Condition + Gender + Form +
+                              (1|Group_Leader) +
+                              (1|Participant_ID:Group:Group_Leader) + (1|School),
+                              data=dfss3, REML=FALSE)
> ## Changing the values in phq4simr to how they were in the data set with missings
> fixef(phq4simr) <- fixef(phqModel_2b)
> sigma(phq4simr) <- sigma(phqModel_2b)
```

```
> VarCorr(phq4simr) <- VarCorr(phqModel_2b)
> phqmodelext.1400 <- extend(phq4simr, along="Participant_ID", n=1400) #we extend along the grouping
>                                         #variable Participant_ID, so we now have 1400 groups = Participants
> phq.ps4<-powerSim(phqmodelext.1400,test=fixed("ConditionStudy-skills",method="t"),
+                   nsim=nsim,alpha=0.045)
> save(phq.ps1,phq.ps2,phq.ps3,phq.ps4,phq.pc1,file="PHQPowerAtMPeffect.rda")
```

```
> phq.ps3 #we have a power of around 98% for 1400 effective sample size (1554 overal) at EP
```

```
Power for predictor 'ConditionStudy-skills', (95% confidence interval):
      98.40% (97.41, 99.08)

Test: t-test with Satterthwaite degrees of freedom (package lmerTest)
      Effect size for ConditionStudy-skills is 1.2

Based on 1000 simulations, (32 warnings, 0 errors)
alpha = 0.045, nrow = 4822

Time elapsed: 0 h 17 m 4 s
```

```
> phq.ps4 #we have a power of around 91% for 1400 effective sample size (1554 overal) at FU
```

```
Power for predictor 'ConditionStudy-skills', (95% confidence interval):
      90.80% (88.84, 92.52)

Test: t-test with Satterthwaite degrees of freedom (package lmerTest)
      Effect size for ConditionStudy-skills is 1.1

Based on 1000 simulations, (38 warnings, 0 errors)
alpha = 0.045, nrow = 4822

Time elapsed: 0 h 14 m 19 s
```

The effective sample size of $n_{eff} = 1400$ ($n^* = 1554$ overall) has an estimated power to detect an effect of $b = -1.24 (d = 0.3)$ at `EP` if all else is equal to the JAMA study of 98%. The effective sample size of $n_{eff} = 1400$ ($n^* = 1554$ overall) has an estimated power to detect an effect of $b = -1.13 (d = 0.28)$ at `FU` if all else is equal to the JAMA study of 91%.

### *Other Target Effect Sizes*

For completeness, we'll also do the calculations with assuming the minimally relevant effect size anywhere to be like at `EP` (roughly like $d = 0.3$) and also with a hypothesized effect difference of $b = 1$ ($d = 0.25$).

**Hypothesized effect of $d \geq 0.3$ everywhere.** First for a hypothesized effect that is always at least as large as at `EP`, so $b = -1.24$.

```
> set.seed(666) #seed for reproducibility
> #re-parametrize so the effect at ConditionStudy-skills is directly the effect we want to detect.
> dfss2$Timef <- relevel(dfss2$Timef,ref="EP")
> dfss3 <- na.omit(dfss2) #remove the missings
> ## original Model refitted
> phqModel_2b <- lmer(PHQsum ~ Timef * Condition + Gender + Form +
+                              (1|Group_Leader) +
+                              (1|Participant_ID:Group:Group_Leader) + (1|School),
+                              data=dfss2, REML = FALSE)
> #model object to use for simr (without missings)
> phq4simr    <- lmer(PHQsum ~ Timef * Condition + Gender + Form +
+                              (1|Group_Leader) +
+                              (1|Participant_ID:Group:Group_Leader) + (1|School),
```

```
+                              data=dfss3, REML=FALSE)
> ## Changing the values in phq4simr to how they were in the data set with missings
> fixef(phq4simr) <- fixef(phqModel_2b)
> sigma(phq4simr) <- sigma(phqModel_2b)
> VarCorr(phq4simr) <- VarCorr(phqModel_2b)
> ## Number of simulated data sets
> nsim <- 500 #we do a bit less simulation due to time
> ## observed power for the 2019 study at MP
> phq.ps1ep<-powerSim(phq4simr,test=fixed("ConditionStudy-skills",method="t"),
+                     nsim=nsim,alpha=0.045)
> ## Create more participants up to a maximum of 1200 (power way over 90%)
> phqmodelext <- extend(phq4simr, along="Participant_ID", n=1200) #we extend along the
>                                          #grouping variable Participant_ID, so we now have 1200 groups =
>                                          #Participants instead of 405 as in the 2019 study
> phq.ps2ep<-powerSim(phqmodelext,test=fixed("ConditionStudy-skills",method="t"),
+                     nsim=nsim,alpha=0.045)
> phq.pc1ep <- powerCurve(phqmodelext,test=fixed("ConditionStudy-skills",method="t"),
+                         along="Participant_ID", nsim=nsim,breaks=c(seq(300,1100,by=100)),
+                         alpha=0.045)
> phq.pc1ep
```

```
Power for predictor 'ConditionStudy-skills', (95% confidence interval),
by number of levels in Participant_ID:
     300: 53.40% (48.92, 57.84) - 1117 rows
     400: 61.40% (56.98, 65.69) - 1412 rows
     500: 72.20% (68.05, 76.09) - 1781 rows
     600: 81.00% (77.28, 84.35) - 2140 rows
     700: 86.00% (82.65, 88.92) - 2531 rows
     800: 88.80% (85.70, 91.43) - 2825 rows
     900: 91.40% (88.59, 93.71) - 3196 rows
    1000: 93.00% (90.40, 95.08) - 3549 rows
    1100: 94.60% (92.24, 96.41) - 3945 rows

Time elapsed: 0 h 28 m 56 s
```

We cross the 80% power boundary for an effect of at least $b = -1.24$ ($d = 0.3$) with an effective sample size of around $n_{eff} = 600$ participants. With the attrition correction factor of $b = 1.11$ this is $n^* = 666$ overall.

For that effective sample size we'd have the following expected power at the hypothesized effects at `MP` and `FU`:

```
> ## FOR MP
> set.seed(666)
> dfss2$Timef <- relevel(dfss2$Timef,ref="MP")
> dfss3 <- na.omit(dfss2) #remove the missings
> ## original Model refitted
> phqModel_2b <- lmer(PHQsum ~ Timef * Condition + Gender + Form +
+                              (1|Group_Leader) +
+                              (1|Participant_ID:Group:Group_Leader) + (1|School),
+                              data=dfss2, REML = FALSE)
> #model object to use for simr (without missings)
> phq4simr    <- lmer(PHQsum ~ Timef * Condition + Gender + Form +
+                              (1|Group_Leader) +
+                              (1|Participant_ID:Group:Group_Leader) + (1|School),
+                              data=dfss3, REML=FALSE)
> ## Changing the values in phq4simr to how they were in the data set with missings
> fixef(phq4simr) <- fixef(phqModel_2b)
> sigma(phq4simr) <- sigma(phqModel_2b)
> VarCorr(phq4simr) <- VarCorr(phqModel_2b)
> phqmodelext.600 <- extend(phq4simr, along="Participant_ID", n=600) #we extend along the grouping
```

```
>                                         #variable Participant_ID, so we now have 600 groups = Participants
> phq.ps3ep<-powerSim(phqmodelext.600,test=fixed("ConditionStudy-skills",method="t"),
+                     nsim=nsim,alpha=0.045)
> ## FOR FU
> dfss2$Timef <- relevel(dfss2$Timef,ref="2WFU")
> dfss3 <- na.omit(dfss2) #remove the missings
> ## original Model refitted
> phqModel_2b <- lmer(PHQsum ~ Timef * Condition + Gender + Form +
+                              (1|Group_Leader) +
+                              (1|Participant_ID:Group:Group_Leader) + (1|School),
+                              data=dfss2, REML = FALSE)
> #model object to use for simr (without missings)
> phq4simr    <- lmer(PHQsum ~ Timef * Condition + Gender + Form +
+                              (1|Group_Leader) +
+                              (1|Participant_ID:Group:Group_Leader) + (1|School),
+                              data=dfss3, REML=FALSE)
> ## Changing the values in phq4simr to how they were in the data set with missings
> fixef(phq4simr) <- fixef(phqModel_2b)
> sigma(phq4simr) <- sigma(phqModel_2b)
> VarCorr(phq4simr) <- VarCorr(phqModel_2b)
> phqmodelext.600 <- extend(phq4simr, along="Participant_ID", n=600) #we extend along the grouping
>                                         #variable Participant_ID, so we now have 1400 groups = Participants
> phq.ps4ep<-powerSim(phqmodelext.600,test=fixed("ConditionStudy-skills",method="t"),
+                     nsim=nsim,alpha=0.045)
> save(phq.ps1ep,phq.ps2ep,phq.ps3ep,phq.ps4ep,phq.pc1ep,file="PHQPowerAtEPeffect.rda")

> #we have a power of around 54% for effective sample size of 600 participants
> #for the mid point effect size (666) overall
> phq.ps3ep

Power for predictor 'ConditionStudy-skills', (95% confidence interval):
      54.00% (49.52, 58.43)

Test: t-test with Satterthwaite degrees of freedom (package lmerTest)
      Effect size for ConditionStudy-skills is -0.89

Based on 500 simulations, (10 warnings, 0 errors)
alpha = 0.045, nrow = 2140

Time elapsed: 0 h 3 m 0 s

> #we have a power of around 60% for effective sample size of 600 participants
> #for the mid point effect size (666) overall
> phq.ps4ep

Power for predictor 'ConditionStudy-skills', (95% confidence interval):
      59.80% (55.35, 64.13)

Test: t-test with Satterthwaite degrees of freedom (package lmerTest)
      Effect size for ConditionStudy-skills is 1.1

Based on 500 simulations, (9 warnings, 0 errors)
alpha = 0.045, nrow = 2140

Time elapsed: 0 h 3 m 36 s
```

For effects of the size as observed at `MP` ($b = 0.89, d = 0.22$) and `FU` ($b = -1.13, d = 0.28$) the power we expect for the effective sample size of $n_{eff} = 600$ (overall $n^* = 666$) are 54% and 60% respectively (around the same power as we had for the effect at `EP` in the 2019 study).

**Hypothesized effect of $n \geq 1$ everywhere.** Now for a hypothesized effect of at least $b = 1(d = 0.25)$ score difference or higher at every time point.

```
> set.seed(666) #seed for reproducibility
> #re-parametrize so the effect at ConditionStudy-skills is directly the effect we want to detect.
> dfss2$Timef <- relevel(dfss2$Timef,ref="EP")
> dfss3 <- na.omit(dfss2) #remove the missings
> ## original Model refitted
> phqModel_2b <- lmer(PHQsum ~ Timef * Condition + Gender + Form +
+                             (1|Group_Leader) +
+                             (1|Participant_ID:Group:Group_Leader) + (1|School),
+                             data=dfss2, REML = FALSE)
> #model object to use for simr (without missings)
> phq4simr    <- lmer(PHQsum ~ Timef * Condition + Gender + Form +
+                             (1|Group_Leader) +
+                             (1|Participant_ID:Group:Group_Leader) + (1|School),
+                             data=dfss3, REML=FALSE)
> ## Changing the values in phq4simr to how they were in the data set with missings
> fixef(phq4simr)["ConditionStudy-skills"] <- 1
> sigma(phq4simr) <- sigma(phqModel_2b)
> VarCorr(phq4simr) <- VarCorr(phqModel_2b)
> ## Number of simulated data sets
> nsim<-500  #again less then before
> ## Create more participants up to a maximum of 1500
> phqmodelext <- extend(phq4simr, along="Participant_ID", n=1500) #we extend along the grouping variable
>                                         #Participant_ID, so we now have 1500 groups = Participants instead
>                                         #of 405 as in the 2019 study
> phq.ps2one<-powerSim(phqmodelext,test=fixed("ConditionStudy-skills",method="t"),
+                       nsim=nsim,alpha=0.045) #we have a power of around 91% for 1500 participants
> phq.pc1one <- powerCurve(phqmodelext,test=fixed("ConditionStudy-skills",method="t"),
+                          along="Participant_ID",nsim=nsim,breaks=c(seq(500,1400,by=100)),
+                          alpha=0.045)
> phq.pc1one

Power for predictor 'ConditionStudy-skills', (95% confidence interval),
by number of levels in Participant_ID:
    500: 48.80% (44.34, 53.28) - 1781 rows
    600: 58.00% (53.54, 62.37) - 2140 rows
    700: 67.60% (63.30, 71.69) - 2531 rows
    800: 72.40% (68.26, 76.28) - 2825 rows
    900: 77.00% (73.06, 80.62) - 3196 rows
   1000: 80.80% (77.07, 84.16) - 3549 rows
   1100: 84.60% (81.13, 87.65) - 3945 rows
   1200: 87.20% (83.95, 90.00) - 4242 rows
   1300: 88.40% (85.26, 91.07) - 4605 rows
   1400: 91.20% (88.37, 93.53) - 4958 rows

Time elapsed: 0 h 46 m 52 s
```

For a hypothesized minimal effect difference at `EP` of $b = -1$ score on the PHQ scale (roughly $d = 0.25$), we cross the 80% power threshold at an effective sample size of $n_{eff} = 1000$ participants ($n^* = 1110$ with attrition correction).

For this effective sample size, we'd have the following estimated power for a hypothesized effect size of $b = 1$ at `MP` and `FU`.

```
> set.seed(666)
> ## FOR MP
> dfss2$Timef <- relevel(dfss2$Timef,ref="MP")
> dfss3 <- na.omit(dfss2) #remove the missings
> ## original Model refitted
> phqModel_2b <- lmer(PHQsum ~ Timef * Condition + Gender + Form +
+                             (1|Group_Leader) +
+                             (1|Participant_ID:Group:Group_Leader) + (1|School),
+                             data=dfss2, REML = FALSE)
```

```
> #model object to use for simr (without missings)
> phq4simr    <- lmer(PHQsum ~ Timef * Condition + Gender + Form +
+                             (1|Group_Leader) +
+                             (1|Participant_ID:Group:Group_Leader) + (1|School),
+                             data=dfss3, REML=FALSE)
> ## Changing the values in phq4simr to how they were in the data set with missings
> fixef(phq4simr)["ConditionStudy-skills"] <- 1
> sigma(phq4simr) <- sigma(phqModel_2b)
> VarCorr(phq4simr) <- VarCorr(phqModel_2b)
> phqmodelext.1000 <- extend(phq4simr, along="Participant_ID", n=1000) #we extend along the grouping
>                                              #variable Participant_ID, so we now have 1000 groups = Participants
> phq.ps3one<-powerSim(phqmodelext.1000,test=fixed("ConditionStudy-skills",method="t"),
+                     nsim=nsim,alpha=0.045)
> ## FOR FU
> dfss2$Timef <- relevel(dfss2$Timef,ref="2WFU")
> dfss3 <- na.omit(dfss2) #remove the missings
> ## original Model refitted
> phqModel_2b <- lmer(PHQsum ~ Timef * Condition + Gender + Form +
+                             (1|Group_Leader) +
+                             (1|Participant_ID:Group:Group_Leader) + (1|School),
+                             data=dfss2, REML = FALSE)
> #model object to use for simr (without missings)
> phq4simr    <- lmer(PHQsum ~ Timef * Condition + Gender + Form +
+                             (1|Group_Leader) +
+                             (1|Participant_ID:Group:Group_Leader) + (1|School),
+                             data=dfss3, REML=FALSE)
> ## Changing the values in phq4simr to how they were in the data set with missings
> fixef(phq4simr)["ConditionStudy-skills"] <- 1
> sigma(phq4simr) <- sigma(phqModel_2b)
> VarCorr(phq4simr) <- VarCorr(phqModel_2b)
> phqmodelext.1000 <- extend(phq4simr, along="Participant_ID", n=1000) #we extend along the grouping
>                                              #variable Participant_ID, so we now have 1000 groups = Participants
> phq.ps4one<-powerSim(phqmodelext.1000,test=fixed("ConditionStudy-skills",method="t"),
+                     nsim=nsim,alpha=0.045)

> phq.ps3one #we have a power of around 82% for 1000 participants at MP.

Power for predictor 'ConditionStudy-skills', (95% confidence interval):
      82.20% (78.56, 85.45)

Test: t-test with Satterthwaite degrees of freedom (package lmerTest)
      Effect size for ConditionStudy-skills is 1.0

Based on 500 simulations, (15 warnings, 0 errors)
alpha = 0.045, nrow = 3549

Time elapsed: 0 h 4 m 8 s

> phq.ps4one #we have a power of around 73% for 1000 participants at FU.

Power for predictor 'ConditionStudy-skills', (95% confidence interval):
      73.00% (68.88, 76.85)

Test: t-test with Satterthwaite degrees of freedom (package lmerTest)
      Effect size for ConditionStudy-skills is 1.0

Based on 500 simulations, (19 warnings, 0 errors)
alpha = 0.045, nrow = 3549

Time elapsed: 0 h 4 m 10 s
```

At `MP` we'd have an estimated power of 82%, at `FU` we'd still have 73%. These differences are due to the differences in standard errors at these times (also related to the attrition that increases over time).

Personally, I think this is a hypothesized effect size that seems practically relevant without being overly optimistic (a small to medium effect of $d = 0.25$). For it we have reasonable power at all time points if we used an effective sample size of $n_{eff} = 1000$ ($n^* = 1110$ participants with attrition correction). As we can see for $n^* = 1110$ the power hovers between 0.73 and 0.83 over all time points which is uniformly relatively high (compare that to the signficant 2019 effect at EP, which had a power of 62% power to be detected). What we have here would be a good compromise between size of the sample, hypothesized effect size and detection power. If we can afford more participants, I'd aim at getting at least 80% power also at $FU$ for an effect of 1; for this we'd need an effective sample size of about $n_{eff} = 1300$ ($n^* = 1443$), see:

```
> #when would we cross 80% for effect of 1 at FU?
> phqmodelext.1500 <- extend(phq4simr, along="Participant_ID", n=1500) #we extend along the grouping
>                                          #variable Participant_ID, so we now have 1500 groups = Participants
> phq.pc2one<-powerCurve(phqmodelext.1500,test=fixed("ConditionStudy-skills",method="t"),
+                        along="Participant_ID", nsim=nsim,breaks=c(seq(1000,1500,by=100)),
+                        alpha=0.045)
> save(phq.ps2one,phq.ps3one,phq.ps4one,phq.pc1one,phq.pc2one,file="PHQPowerAtOne.rda")

> phq.pc2one

Power for predictor 'ConditionStudy-skills', (95% confidence interval),
by number of levels in Participant_ID:
   1000: 70.20% (65.98, 74.18) - 3549 rows
   1100: 75.80% (71.80, 79.49) - 3945 rows
   1200: 77.80% (73.90, 81.37) - 4242 rows
   1300: 81.80% (78.13, 85.09) - 4605 rows
   1400: 84.80% (81.35, 87.83) - 4958 rows
   1500: 86.80% (83.51, 89.64) - 5355 rows

Time elapsed: 0 h 32 m 8 s
```

## 4.3. GAD

For GAD we observed significant differences between Wellness (the Shamiri treatment condition) and Study-Skills (the control condition) at EP. We did not find a significant effect at 2WFUP (but barely). At MP the effect size is so low that it is of no practical consequence ($d < 0.2$); I'll therefore ignore MP when doing the calculations for GAD. These results are repeated subsequently with REML=FALSE. The fitted model was (now with REML=FALSE).

```
> ## full specification of the nesting as I think it should be - explicit
> gadModel_2b <- lmer(GADsum ~ Timef * Condition + Gender + Form +
+                         (1|Group_Leader) + (1|Group:Group_Leader) +
+                         (1|Participant_ID:Group:Group_Leader) + (1|School),
+                     data=dfss2, REML = FALSE, verbose=0)
> summary(gadModel_2b)

Linear mixed model fit by maximum likelihood . t-tests use Satterthwaite's
  method [lmerModLmerTest]
Formula: GADsum ~ Timef * Condition + Gender + Form + (1 | Group_Leader) +
    (1 | Group:Group_Leader) + (1 | Participant_ID:Group:Group_Leader) +
    (1 | School)
   Data: dfss2

     AIC      BIC   logLik deviance df.resid
  8282.9   8383.1  -4122.4   8244.9     1427

Scaled residuals:
    Min      1Q  Median      3Q     Max
-2.8459 -0.6452 -0.0978  0.5660  3.2302
```

```
Random effects:
 Groups                           Name        Variance Std.Dev.
 Participant_ID:Group:Group_Leader (Intercept)  4.27064 2.0666
 Group:Group_Leader               (Intercept)  0.18633 0.4317
 Group_Leader                     (Intercept)  0.08585 0.2930
 School                           (Intercept)  0.00000 0.0000
 Residual                                     14.25033 3.7750
Number of obs: 1446, groups:
Participant_ID:Group:Group_Leader, 409; Group:Group_Leader, 45; Group_Leader, 15; School, 4

Fixed effects:
                                  Estimate Std. Error        df t value
(Intercept)                      6.389e+00  4.736e-01  1.963e+02  13.491
TimefBL                          6.705e+00  4.223e-01  1.130e+03  15.876
TimefMP                          2.993e+00  4.587e-01  1.117e+03   6.526
Timef2WFU                       -1.235e+00  4.858e-01  1.144e+03  -2.543
Timef7MFU                        3.465e-01  4.885e-01  1.172e+03   0.709
ConditionStudy-skills            1.674e+00  5.211e-01  2.534e+02   3.212
GenderFemale                     2.852e-01  3.548e-01  6.327e+01   0.804
Form2                           -9.114e-03  3.406e-01  3.758e+02  -0.027
Form3                            2.863e-01  4.221e-01  4.133e+02   0.678
Form4                           -3.654e-01  7.005e-01  3.375e+02  -0.522
TimefBL:ConditionStudy-skills   -1.775e+00  5.870e-01  1.126e+03  -3.024
TimefMP:ConditionStudy-skills   -2.180e+00  6.312e-01  1.112e+03  -3.454
Timef2WFU:ConditionStudy-skills -6.181e-01  6.794e-01  1.143e+03  -0.910
Timef7MFU:ConditionStudy-skills  5.682e-01  6.918e-01  1.163e+03   0.821
                                Pr(>|t|)
(Intercept)                      < 2e-16 ***
TimefBL                          < 2e-16 ***
TimefMP                         1.02e-10 ***
Timef2WFU                       0.011119 *
Timef7MFU                       0.478319
ConditionStudy-skills           0.001487 **
GenderFemale                    0.424478
Form2                           0.978669
Form3                           0.497971
Form4                           0.602253
TimefBL:ConditionStudy-skills   0.002549 **
TimefMP:ConditionStudy-skills   0.000574 ***
Timef2WFU:ConditionStudy-skills 0.363150
Timef7MFU:ConditionStudy-skills 0.411681
---
Signif. codes:  0 âĂŸ***âĂŹ 0.001 âĂŸ**âĂŹ 0.01 âĂŸ*âĂŹ 0.05 âĂŸ.âĂŹ 0.1 âĂŸ âĂŹ 1
optimizer (nloptwrap) convergence code: 0 (OK)
boundary (singular) fit: see help('isSingular')
```

and the condition differences at each time point were

```
> lsm2 <- ls_means(gadModel_2b,which="Timef:Condition",pairwise=TRUE)
> lsm2[c("TimefMP:ConditionWellness - TimefMP:ConditionStudy-skills",
+        "TimefEP:ConditionWellness - TimefEP:ConditionStudy-skills",
+        "Timef2WFU:ConditionWellness - Timef2WFU:ConditionStudy-skills"),]
```

```
Least Squares Means table:

                                                              Estimate
TimefMP:ConditionWellness - TimefMP:ConditionStudy-skills      0.506259
TimefEP:ConditionWellness - TimefEP:ConditionStudy-skills     -1.673843
Timef2WFU:ConditionWellness - Timef2WFU:ConditionStudy-skills -1.055791
                                                              Std. Error    df
TimefMP:ConditionWellness - TimefMP:ConditionStudy-skills       0.522128 256.8
TimefEP:ConditionWellness - TimefEP:ConditionStudy-skills       0.521071 253.4
Timef2WFU:ConditionWellness - Timef2WFU:ConditionStudy-skills   0.575820 367.7
                                                              t value     lower
TimefMP:ConditionWellness - TimefMP:ConditionStudy-skills      0.9696 -0.521940
TimefEP:ConditionWellness - TimefEP:ConditionStudy-skills     -3.2123 -2.700024
Timef2WFU:ConditionWellness - Timef2WFU:ConditionStudy-skills -1.8335 -2.188105
                                                                  upper
TimefMP:ConditionWellness - TimefMP:ConditionStudy-skills      1.534458
TimefEP:ConditionWellness - TimefEP:ConditionStudy-skills     -0.647662
Timef2WFU:ConditionWellness - Timef2WFU:ConditionStudy-skills  0.076524
                                                              Pr(>|t|)
TimefMP:ConditionWellness - TimefMP:ConditionStudy-skills     0.333155
TimefEP:ConditionWellness - TimefEP:ConditionStudy-skills     0.001487 **
Timef2WFU:ConditionWellness - Timef2WFU:ConditionStudy-skills 0.067530 .
---
Signif. codes:  0 âĂŸ***âĂŹ 0.001 âĂŸ**âĂŹ 0.01 âĂŸ*âĂŹ 0.05 âĂŸ.âĂŹ 0.1 âĂŸ âĂŹ 1

  Confidence level: 95%
  Degrees of freedom method: Satterthwaite
```

That smallest effect is the effect of $b = -0.5$ at `MP` (see above). This roughly corresponds to a standardized effect size of $d = 0.13$ (dividing the effect by the residual standard error

from the model above). This is an effect that is too low to be of practical relevance and would need a really high $n_{eff}$, so I'll ignore `MP` and its size even though it was said we're also interested in `MP` (but there really is no point in an effect of $d = 0.15$). I'll therefore be running the simulation with `FU` as the minimum effect size, which was $b = -1.05$ (corresponding to $d = 0.28$): I'll also check power for the effect size that corresponds to the effect at `EP` but since that one is larger we will have more power anyway for the effective sample size obtained for `FU` (or would need less $n$ for the same power).

In a nutshell therefore: The sample size determined with $b = -1.05$ will be the minimum sample size we need to detect *any* of our observed effects at `EP` or `FU` with power of 80% in 2019 for GAD. Also note that at `EP` the observed effect corresponds to $d = 0.45$, which is by far the largest standardized effect size we have. Since we will collect GAD and PHQ data for the same people, we only have one sample size $n^*$ that we will use and the question will be whether this is the one for `MP` of PHQ or `FU` for GAD—whichever is largest will be the $n^*$ we need to use.

```
> set.seed(666) #seed for reproducibility
> #re-parametrize so the effect at ConditionStudy-skills is directly the effect we want to detect.
> dfss2$Timef <- relevel(dfss2$Timef,ref="2WFU")
> dfss3 <- na.omit(dfss2) #remove the missings as otherwise simr::powerSim will only return errors
> ## original Model refitted
> gadModel_2b <- lmer(GADsum ~ Timef * Condition + Gender + Form +
+                               (1|Group_Leader) +  (1|Group:Group_Leader) +
+                               (1|Participant_ID:Group:Group_Leader) + (1|School),
+                               data=dfss2, REML = FALSE)
> #model object to use for simr (without missings)
> gad4simr    <- lmer(GADsum ~ Timef * Condition + Gender + Form +
+                               (1|Group_Leader) +  (1|Group:Group_Leader) +
+                               (1|Participant_ID:Group:Group_Leader) + (1|School),
+                               data=dfss3, REML=FALSE)
> ## Changing the values in gad4simr to how they were in the JAMA data set with missings
> fixef(gad4simr) <- fixef(gadModel_2b)
> sigma(gad4simr) <- sigma(gadModel_2b)
> VarCorr(gad4simr) <- VarCorr(gadModel_2b)
> ## Number of simulated data sets
> nsim<-1000
> #### Power Curve Calculation
> ## Create more participants up to a maximum of 2000 that we then cut back (this is how simr works)
> gadmodelext <- extend(gad4simr, along="Participant_ID", n=2000) #we extend along the grouping
>                                         #variable Participant_ID, so we now have 2000 Participants
>                                         #instead of 405 as in the 2019 study
> gad.ps2<-powerSim(gadmodelext,test=fixed("ConditionStudy-skills",method="t"),
+                   nsim=nsim,alpha=0.045) #93% power for 2000 participants
> gad.ps2 #we have a power of around 93% for 2000 participants (with replication of the attrition)
> ## Power curve. Where we cross the 80% threshold is where we have determined the necessary sample size
> gad.pc1 <- powerCurve(gadmodelext,test=fixed("ConditionStudy-skills",method="t"),
+                       along="Participant_ID", nsim=nsim,breaks=c(seq(500,1900,by=100)),
+                       alpha=0.045)
> gad.pc1
```

```
Power for predictor 'ConditionStudy-skills', (95% confidence interval),
by number of levels in Participant_ID:
    500: 53.30% (50.15, 56.43) - 1786 rows
    600: 60.80% (57.70, 63.84) - 2141 rows
    700: 66.80% (63.78, 69.72) - 2528 rows
    800: 70.30% (67.36, 73.12) - 2828 rows
    900: 74.50% (71.68, 77.18) - 3203 rows
   1000: 77.60% (74.89, 80.15) - 3551 rows
```

```
1100: 82.70% (80.21, 85.00) - 3943 rows
1200: 84.40% (82.00, 86.60) - 4245 rows
1300: 86.60% (84.33, 88.65) - 4613 rows
1400: 88.80% (86.68, 90.69) - 4961 rows
1500: 90.10% (88.08, 91.88) - 5355 rows
1600: 92.30% (90.47, 93.88) - 5656 rows
1700: 93.30% (91.57, 94.77) - 6026 rows
1800: 93.90% (92.23, 95.30) - 6376 rows
1900: 94.80% (93.24, 96.09) - 6765 rows

Time elapsed: 3 h 24 m 16 s
```

We cross the 80% power boundary for an effect of at least $b = 1.05$ ($d = 0.28$) with around $n_{eff} = 1100$ participants. With the attrition correction factor of 1.11 this is $n^* = 1221$.

What power would we have for the test at `EP` and `FU`? (we use 1100 as that is the effective sample size)

```
> set.seed(666)
> ## FOR EP
> dfss2$Timef <- relevel(dfss2$Timef,ref="EP")
> dfss3 <- na.omit(dfss2) #remove the missings
> ## original Model refitted
> gadModel_2b <- lmer(GADsum ~ Timef * Condition + Gender + Form +
+                             (1|Group_Leader) +  (1|Group:Group_Leader) +
+                             (1|Participant_ID:Group:Group_Leader) + (1|School),
+                             data=dfss2, REML = FALSE)
> #model object to use for simr (without missings)
> gad4simr    <- lmer(GADsum ~ Timef * Condition + Gender + Form +
+                             (1|Group_Leader) +  (1|Group:Group_Leader) +
+                             (1|Participant_ID:Group:Group_Leader) + (1|School),
+                             data=dfss3, REML=FALSE)
> ## Changing the values in gad4simr to how they were in the data set with missings
> fixef(gad4simr) <- fixef(gadModel_2b)
> sigma(gad4simr) <- sigma(gadModel_2b)
> VarCorr(gad4simr) <- VarCorr(gadModel_2b)
> gadmodelext.1100 <- extend(gad4simr, along="Participant_ID", n=1100) #we extend along the grouping
>                                           #variable Participant_ID, so we now have 1100 groups = Participants
> gad.ps3<-powerSim(gadmodelext.1100,test=fixed("ConditionStudy-skills",method="t"),
+                  nsim=nsim,alpha=0.045)
> gad.ps3 #we have a power of around 100% for 1100 participants at EP
> save(gad.ps2,gad.ps3,gad.pc1,file="GADPowerAtFUeffect.rda")


> gad.ps3 #we have a power of around 100% for 1100 effective sample size (1221 overall) at EP


Power for predictor 'ConditionStudy-skills', (95% confidence interval):
      99.80% (99.28, 99.98)

Test: t-test with Satterthwaite degrees of freedom (package lmerTest)
      Effect size for ConditionStudy-skills is 1.7

Based on 1000 simulations, (47 warnings, 0 errors)
alpha = 0.045, nrow = 3943

Time elapsed: 0 h 15 m 39 s
```

The effective sample size of $n_{eff} = 1100$ ($n^* = 1221$ overall) has an estimated power to detect the effect of $b = -1.7$ at `EP` if all else is equal to the JAMA study of 99.8%. The effective sample size of $n_{eff} = 1100$ ($n^* = 1221$ overall) has an estimated power to detect an effect of $b = -1.05$ at `FU` if all else is equal to the JAMA study of 80%. Since 1221 is smaller than 1554 I'd go with the PHQ one.

*Other Target Effect Sizes*

For completeness, we'll also do the calculations with assuming the minimally relevant effect size to be a hypothesized effect difference of $b = -0.95$ ($d = 0.25$).

**Hypothesized effect of $d \geq 0.25$ everywhere.** Now for a hypothesized effect of at least $b = 0.95$ score difference or higher at every time point.

```
> set.seed(666) #seed for reproducibility
> #re-parametrize so the effect at ConditionStudy-skills is directly the effect we want to detect.
> dfss2$Timef <- relevel(dfss2$Timef,ref="EP")
> dfss3 <- na.omit(dfss2) #remove the missings
> ## original Model refitted
> gadModel_2b <- lmer(GADsum ~ Timef * Condition + Gender + Form +
+                              (1|Group_Leader) +  (1|Group:Group_Leader) +
+                              (1|Participant_ID:Group:Group_Leader) + (1|School),
+                              data=dfss2, REML = FALSE)
> #model object to use for simr (without missings)
> gad4simr    <- lmer(GADsum ~ Timef * Condition + Gender + Form +
+                              (1|Group_Leader) +  (1|Group:Group_Leader) +
+                              (1|Participant_ID:Group:Group_Leader) + (1|School),
+                              data=dfss3, REML=FALSE)
> ## Changing the values in gad4simr to how they were in the data set with missings
> fixef(gad4simr)["ConditionStudy-skills"] <- 0.95
> sigma(gad4simr) <- sigma(gadModel_2b)
> VarCorr(gad4simr) <- VarCorr(gadModel_2b)
> ## Number of simulated data sets
> nsim<-500  #again less then before
> ## Create more participants up to a maximum of 1500
> gadmodelext <- extend(gad4simr, along="Participant_ID", n=1500) #we extend along the grouping
>                                         #variable Participant_ID, so we now have 1500 groups = Participants
>                                         #instead of 405 as in the 2019 study
> gad.ps2one<-powerSim(gadmodelext,test=fixed("ConditionStudy-skills",method="t"),
+                      nsim=nsim,alpha=0.045) #we have a power of around 91% for 1500 participants
> gad.pc1one <- powerCurve(gadmodelext,test=fixed("ConditionStudy-skills",method="t"),
+                          along="Participant_ID", nsim=nsim,breaks=c(seq(500,1400,by=100)),
+                          alpha=0.045)
> save(gad.ps2one,gad.pc1one,file="GADPowerAtOne.rda")
> gad.pc1one

Power for predictor 'ConditionStudy-skills', (95% confidence interval),
by number of levels in Participant_ID:
    500: 51.20% (46.72, 55.66) - 1786 rows
    600: 58.20% (53.74, 62.56) - 2141 rows
    700: 63.00% (58.60, 67.24) - 2528 rows
    800: 68.60% (64.33, 72.65) - 2828 rows
    900: 73.40% (69.30, 77.22) - 3203 rows
   1000: 78.00% (74.11, 81.56) - 3551 rows
   1100: 80.00% (76.22, 83.42) - 3943 rows
   1200: 81.60% (77.92, 84.90) - 4245 rows
   1300: 84.20% (80.70, 87.29) - 4613 rows
   1400: 87.20% (83.95, 90.00) - 4961 rows

Time elapsed: 1 h 16 m 52 s
```

For a hypothesized minimal effect difference at `EP` of $b = -0.95$ score on the GAD scale (roughly $d = 0.25$), we cross the 80% power threshold at an effective sample size of $n_{eff} = 1100$ participants ($n^* = 1221$ with attrition correction). This is the same as above because at `EP` we had less attrition (so larger sample and more power) which offsets the larger effect at `2WFU` that had more attrition (so smaller sample and less power).

## 4.4. Computational Info

This computational setup was used for the calculations above.

```
> sessionInfo()
```

```
R version 4.2.3 (2023-03-15)
Platform: x86_64-pc-linux-gnu (64-bit)
Running under: Linux Mint 19.2

Matrix products: default
BLAS:   /usr/lib/x86_64-linux-gnu/blas/libblas.so.3.7.1
LAPACK: /usr/lib/x86_64-linux-gnu/lapack/liblapack.so.3.7.1

locale:
 [1] LC_CTYPE=en_US.UTF-8       LC_NUMERIC=C
 [3] LC_TIME=en_US.UTF-8        LC_COLLATE=en_US.UTF-8
 [5] LC_MONETARY=de_AT.UTF-8    LC_MESSAGES=en_US.UTF-8
 [7] LC_PAPER=de_AT.UTF-8       LC_NAME=C
 [9] LC_ADDRESS=C               LC_TELEPHONE=C
[11] LC_MEASUREMENT=de_AT.UTF-8 LC_IDENTIFICATION=C

attached base packages:
[1] stats     graphics  grDevices utils     datasets  methods   base

other attached packages:
[1] simr_1.0.6     sjPlot_2.8.12  effects_4.2-2  carData_3.0-5  dplyr_1.1.0
[6] lmerTest_3.1-3 lme4_1.1-31    Matrix_1.5-3

loaded via a namespace (and not attached):
 [1] Rcpp_1.0.10         mvtnorm_1.1-3       lattice_0.20-45
 [4] tidyr_1.3.0         zoo_1.8-11          utf8_1.2.3
 [7] plyr_1.8.8          R6_2.5.1            backports_1.4.1
[10] survey_4.1-1        coda_0.19-4         ggplot2_3.4.1
[13] pillar_1.8.1        rlang_1.0.6         multcomp_1.4-22
[16] minqa_1.2.5         performance_0.10.2  car_3.1-1
[19] nloptr_2.0.3        ggeffects_1.2.0     splines_4.2.3
[22] stringr_1.5.0       munsell_0.5.0       broom_1.0.3
[25] compiler_4.2.3      numDeriv_2016.8-1.1 modelr_0.1.10
[28] xfun_0.37           pkgconfig_2.0.3     mgcv_1.8-42
[31] mitools_2.4         nnet_7.3-18         insight_0.19.0
[34] tidyselect_1.2.0    tibble_3.1.8        binom_1.1-1.1
[37] codetools_0.2-19    fansi_1.0.4         withr_2.5.0
[40] MASS_7.3-58.3       sjmisc_2.8.9        grid_4.2.3
[43] nlme_3.1-162        xtable_1.8-4        gtable_0.3.1
[46] lifecycle_1.0.3     DBI_1.1.3           magrittr_2.0.3
[49] bayestestR_0.13.0   scales_1.2.1        datawizard_0.6.5
[52] stringi_1.7.12      estimability_1.4.1  cli_3.6.0
[55] generics_0.1.3      vctrs_0.5.2         boot_1.3-28.1
[58] sandwich_3.0-2      sjlabelled_1.2.0    TH.data_1.1-1
[61] iterators_1.0.14    tools_4.2.3         glue_1.6.2
[64] purrr_1.0.1         sjstats_0.18.2      emmeans_1.8.4-1
[67] plotrix_3.8-2       parallel_4.2.3      pbkrtest_0.5.2
[70] abind_1.4-5         survival_3.5-5      colorspace_2.1-0
[73] RLRsim_3.1-8        knitr_1.42
```

## Affiliation:

Thomas Rusch
Competence Center for Empirical Research Methods
WU Vienna (Wirtschaftsuniversität Wien)
Welthandelsplatz 1, D5
1020 Wien, Austria
E-mail: Thomas.Rusch@wu.ac.at
